# Diversity of the Genus *Xylaria* in European Atlantic Lauroid Forest: New Records and Description of Eight New Species

**DOI:** 10.3390/life16060993

**Published:** 2026-06-12

**Authors:** Saúl De la Peña-Lastra, Antonio Mateos, Abelardo García-Martín, Antonio Rigueiro-Rodríguez, Miguel Serrano

**Affiliations:** 1 Ecology Section, Faculty of Biology, Universidade de Santiago de Compostela, 15782 Santiago de Compostela, Spain; 2 Sociedad Micológica Extremeña, 10001 Cáceres, Spain; 3 Department of Agricultural and Forestry Engineering, School of Agricultural Engineering, Universidad de Extremadura, 06007 Badajoz, Spain; 4 Department of Crop Production and Engineering Projects, Escuela Politécnica Superior de Lugo, Universidade de Santiago de Compostela, Campus Universitario s/n, 27002 Lugo, Spain; 5 Department of Botany, Faculty of Pharmacy, Universidade de Santiago de Compostela, 15782 Santiago de Compostela, Spain; miguel.serrano@usc.es

**Keywords:** *Xylaria*, Xylariaceae, diversity hotspot, phylogeny, macrofungi, laurel forest, laurisilva

## Abstract

The genus *Xylaria* (Xylariaceae, Ascomycota) comprises a morphologically and ecologically diverse group of fungi with a predominantly saprobic lifestyle, widely distributed in forest ecosystems worldwide. Despite its global occurrence, its diversity in European Atlantic laurel forests (laurisilva), both insular and continental, remains poorly understood. In this study, we examined more than 80 collections of *Xylaria* from laurisilva forests in Madeira and the Azores (Portugal), the Canary Islands (Spain), and relict laurel woodlands in mainland Iberia, documenting at least 13 species. Several collections could not be successfully sequenced, suggesting that additional taxa may occur. Among the identified species, eight are described here as new to science and are supported by morphological differences and multilocus phylogenetic analyses. Species delimitation was based on an integrative approach combining detailed morphological observations with phylogenetic inference from ITS, LSU, *RPB2*, and *TUB2* loci. Our results reveal a substantially higher diversity of *Xylaria* in these ecosystems than previously recognized and confirm the importance of multilocus frameworks for resolving species boundaries, particularly in morphologically cryptic lineages. This study expands the known diversity of *Xylaria* in Europe and identifies Atlantic laurel forests as important reservoirs of fungal diversity and evolutionary novelty.

## 1. Introduction

The genus *Xylaria* (Xylariaceae, Ascomycota) represents one of the most diverse groups of ascomycetous fungi, comprising approximately 300 species [1]. These fungi are primarily known for their capacity to degrade wood and for their widespread occurrence in forest habitats worldwide (e.g., [2,3,4]). Species of *Xylaria* play a crucial role in nutrient cycling through the decomposition of lignocellulosic material, but may also occur as endophytes or, less frequently, as pathogens in a wide range of host plants [5]. This ecological versatility, together with their high morphological and genetic diversity, makes the genus an important component of fungal biodiversity in forest ecosystems.

Although the genus has been extensively studied in Europe (e.g., [6,7,8,9,10,11]) and in tropical and subtropical regions, such as South America, Mexico, and the Caribbean (including Guadeloupe and Martinique, e.g., [2,5,12,13,14,15,16], Southeast Asia, e.g., [3,17,18], and Africa e.g., [19,20,21]), its diversity in specific habitats such as the Atlantic laurel forests remains poorly understood. Laurisilva forests, characterized by high humidity, moderate temperatures, and the dominance of evergreen laurophyllous trees, provide favorable conditions for lignicolous fungi. Despite these favorable conditions, available studies in these forest formations, both in their insular components (Madeira and the Canary Islands) and in continental remnants of the Iberian Peninsula, remain relatively scarce (e.g., [22,23,24,25]).

Tropical studies have consistently revealed that the diversity of *Xylaria* in humid forest ecosystems is substantially higher than previously assumed, particularly when intensive and systematic sampling strategies are applied. Surveys conducted in Caribbean rainforests have documented a wide assemblage of species associated with decaying wood, including both pantropical taxa and numerous morphologically similar or poorly resolved entities. Notably, these investigations suggest a strong detection bias toward species with robust and conspicuous stromata, whereas a significant fraction of the diversity is represented by more delicate or inconspicuous forms that remain under-sampled and taxonomically unresolved, suggesting that current inventories likely underestimate the true species richness of the genus [14].

In parallel, studies in Neotropical montane forests have emphasized the persistent taxonomic complexity of *Xylaria* and the limitations of traditional identification approaches in resolving species boundaries. Integrative analyses combining morphology and DNA barcoding in Andean ecosystems have revealed the coexistence of multiple phylogenetically distinct lineages within restricted spatial scales, even in areas subject to previous mycological exploration. These findings highlight the prevalence of cryptic diversity and reinforce the need for multilocus frameworks to accurately delimit species, supporting the hypothesis that structurally complex and humid environments, such as Atlantic laurel forests, may harbor a substantially underestimated diversity of *Xylaria* [26].

From a biogeographical perspective, Atlantic laurel forests are considered relict ecosystems that preserve elements of the ancient Tertiary flora, acting as long-term refugia for numerous plant and fungal lineages. Their ecological stability, combined with geographic isolation in island systems, such as Madeira, the Azores, and the Canary Islands, as well as their fragmented distribution in continental areas, creates conditions that may promote diversification and endemism [27]. In this context, these forests likely harbor unique fungal assemblages, including potentially endemic or highly specialized species of *Xylaria*, with a significant proportion likely remaining undocumented or poorly characterized.

Several previous observations have documented the presence of species of *Xylaria* in these laurel-dominated environments, including taxa such as *Xylaria arbuscula* and *Xylaria apiculata* in the Canary Islands and in the island of Cortegada off the coast of the Iberian Peninsula [11,28,29], *Xylaria cinerea*, *Xylaria violaceorosea*, and *X. digitata* in Cortegada [29], as well as *Xylaria polymorpha* and *X. hypoxylon*, cosmopolitan species recorded in these forests of the Canary Islands, Madeira, Cortegada, and continental areas, adapted to different types of decaying wood [25,28,29,30]. However, beyond these general records, evidence points to the occurrence of additional, potentially endemic or insufficiently resolved taxa in these habitats, suggesting a higher diversity than currently recognized.

From a systematic perspective, species of *Xylaria* have been included in numerous phylogenetic studies within Xylariaceae. However, most of these studies have relied exclusively or predominantly on the analysis of the ITS region of ribosomal DNA [31,32,33,34] or have combined ITS with other ribosomal regions and/or protein-coding genes [35,36,37]. Several studies have demonstrated that the exclusive use of ITS is insufficient to adequately resolve phylogenetic relationships within *Xylaria*, particularly in complexes of morphologically cryptic species. In this regard, multigene analyses have proven essential for robust taxonomic delimitation within Xylariaceae. This was clearly demonstrated in the study of Wendt et al. [38], which, based on the combination of multiple loci (ITS, LSU, *RPB2*, and *TUB2*), enabled a substantial restructuring of the principal lineages within Xylariales.

In this context, our integrative study provides important new insights into the diversity of the genus *Xylaria* in European Atlantic laurel forests. Through extensive sampling across Madeira, the Azores, the Canary Islands, and relict laurel forests of the Iberian Peninsula, using an integrative approach combining morphological and molecular analyses, we identified several previously undocumented *Xylaria* species in these ecosystems.

Our results highlight the importance of Atlantic laurel forests as refugia of fungal diversity and emphasize the need for continued mycological exploration of these unique ecosystems, not only to document existing biodiversity but also to improve our understanding of the ecological dynamics in which lignicolous fungi such as *Xylaria* play a fundamental role.

## 2. Materials and Methods

### 2.1. Study Area and Sampling Design

**Figure 1:**
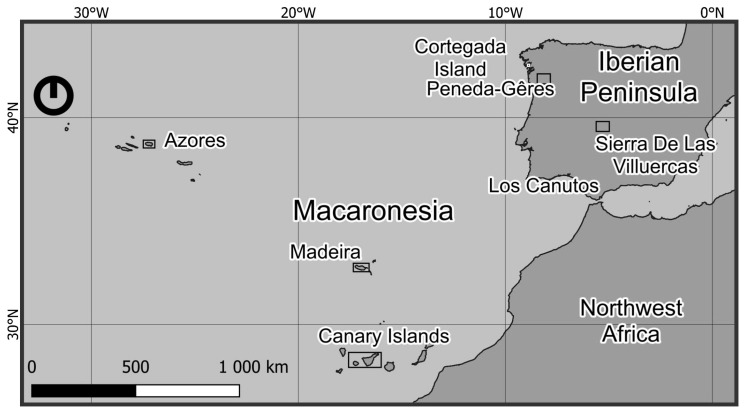
Geographical location of the study areas across Macaronesia and the Iberian Peninsula. Sampling was carried out in Atlantic laurel forests of the Azores, Madeira, and the Canary Islands, as well as in relict laurel forest remnants of Cortegada Island, Peneda-Gerês, Sierra de las Villuercas, and Los Canutos. The symbol in the upper-left corner indicates geographic north.

This study was carried out in Atlantic lauroid forest ecosystems distributed across both insular and continental regions, encompassing some of the best-preserved remnants of this Tertiary vegetation type (Figure 1 and Table 1).

All required permits were obtained from the managing authorities of the protected areas prior to fieldwork, and all sampling activities were carried out in compliance with biodiversity legislation in Spain and Portugal.

A total of ten laurel forest sites were sampled, including seven insular sites, six of which were located in Macaronesia, and three continental sites in the Iberian Peninsula. Each locality was sampled during the wet season (autumn and winter) in the years 2021–2024. Within each locality three sampling areas were selected based on the presence of well-developed laurel forest stands dominated by one or two characteristic lauroid tree species, including *Laurus nobilis* L., *Laurus azorica* (Seub.) Franco, or *Laurus novocanariensis* Rivas Mart., Lousã, Fern.Prieto, E.Días, J.C.Costa & C.Aguiar, which are genetically closely related as well as subspecies of *Prunus lusitanica* L. At each sampling area, standardized surveys were conducted for three hours per dominant tree species, with one hour devoted to sampling fungi growing on soil, one hour on woody substrates, and one hour on leaf litter.

### 2.2. Morphological and Microscopic Analyses

Macrofungal specimens were photographed in situ using Nikon D-90 (Nikon, Tokyo, Japan) and Canon EOS 7D Mark II digital cameras (Canon, Tokyo, Japan) equipped with a Canon EF 100 mm f/2.8 Macro USM lens. Macroscopic and microscopic descriptions were conducted on fresh material. Microscopic examinations were performed on living specimens using an OPTIKA B-383 PLI light microscope (OPTIKA, Optika, Italy) with an oil immersion objective up to ×1000, coupled with a Canon EOS 1300 camera for microphotography, while an OPTIKA SZM-1 stereomicroscope was employed for sample preparation and macroscopic detail photographs. Microscopic observations were made in water, 2% KOH, Melzer’s reagent, and IKI 2 (to test the amyloidity of the ascus apical apparatus), ascospores were mounted in diluted India ink to show the secondary appendages, and aqueous Congo Red SDS for general staining, and spores were examined in a living hydrated state following the principles of vital taxonomy [39]. Digital images were subsequently processed using Adobe Photoshop 23.5.2 and Helicon Focus 8.1.0 (2022). Micromorphological measurements were obtained with Piximètre 5.9 software [40], calibrated for each objective, and spore size and shape descriptions followed the criteria established by Bas [41].

All examined material is deposited in the private herbarium of Antonio Mateos (AMI-Spain). Species nomenclature and taxonomic classification follow the standards proposed by Index Fungorum and MycoBank, in conjunction with updated monographs, original species descriptions, and relevant scientific literature.

### 2.3. DNA Extraction and Amplification

Total DNA was extracted from silica-gel-dried samples or from herbarium specimens using the NucleoSpin Plant II kit (Macherey-Nagel, Düren, Germany) or the Animal and Fungi DNA Preparation Kit (Jena, Thuringia, Germany). DNA quantity and quality were assessed with a Nanodrop 2000c spectrophotometer (Thermo Scientific, Waltham, MA, USA). Four genomic regions were amplified by PCR using a PxE02 thermocycler (Thermoelectron, Waltham, MA, USA) or Veriti 96-Well Thermal Cycling (Applied Biosystems, Waltham, MA, USA). The ITS region was amplified with the primer pair ITS5 (5′-GGAAGGAGAAGTCGTAACAAGG-3′) and ITS4 (5′-TCCTCCGCTTATTGATATGC-3′) [42], and PCR conditions were 1 cycle of 95 °C/3 min, plus 34 cycles of 94 °C/30 s, 54 °C/30 s, 72 °C/2 min, and a final extension cycle of 72 °C/10 min. The nLSU (large subunit of ribosomal DNA) used the primer pair LROR (5′-GTACCCGCTGAACTTAAGC) [43] and LR7 (5′-TACTACCACCAAGATCT) [44], and PCR conditions were 1 cycle of 95 °C/5 min, plus 35 cycles of 94 °C/30 s, 52 °C/30 s, 72 °C/90 s, and a final extension cycle of 72 °C/8 min. The rpb2 gene used the primer pair RPB2-5f (5′-GAYGAYMGWGATCAYTTYGG-3′) and RPB2-7cR (5′-CCCATRGCTTGTYYRCCCAT-3′) [45], and in some cases the primer fRPB2-5F2 (5′-GGGGWGAYCAGAAGAAGGC), following recommendations of Sung et al. [46], and PCR conditions were 1 cycle of 94 °C/5 min, plus 35 cycles of 94 °C/60 s, 57 °C/60 s, 72 °C/90 s, and a final extension cycle of 72 °C/10 min. Other annealing temperatures were tested (between 52 °C and 55 °C) in those samples that failed to amplify. The β-Tubulin gene used the primer pair T1 (5′-AACATGCGTGAGATTGTAAGT-3′) [47] and Bt2b (5′-ACCCTCAGTGTAGTGACCCTTGGC-3′) [48], and PCR conditions were 1 cycle of 95 °C/5 min, plus 35 cycles of 94 °C/45 s, 55 °C/45 s, 72 °C/60 s, and a final extension cycle of 72 °C/7 min. Sanger sequencing of the ITS region was carried out with an ABI 3730 xl sequencer (Applied Biosystems) by STAB VIDA, Portugal, while sequencing of the LSU, rpb2, and β-Tubulin regions was conducted with an ABI PRISM 3730 sequencer (Applied Biosystems) by the SCSIE of the University of Valencia, Spain. The resulting sequences were deposited in GenBank with the accession numbers listed in Table 2.

### 2.4. Phylogenetic Analysis

Sequence datasets were prepared for each gene region by combining the sequences generated in this study and additional sequences retrieved from GenBank. Given the large number of species currently recognized in *Xylaria*, taxon sampling was designed to include those species most relevant for assessing the phylogenetic placement of the studied collections. Sequences were selected based on three complementary criteria: (i) phylogenetic affinity inferred from preliminary BLASTn searches against GenBank using the newly generated sequences, (ii) inclusion of morphologically similar taxa and species previously reported from Europe, and (iii) availability of sequence data for the loci analyzed (ITS, LSU, *RPB2*, and *TUB2*). This strategy resulted in a representative dataset while minimizing missing data and maximizing phylogenetic resolution for species delimitation purposes. Given that amplification success varied among gene regions and specimens, the concatenated dataset contained some missing sequence data (Table 2). Nevertheless, all taxa were represented by ITS sequences, and all additional loci successfully obtained were incorporated into the analyses to maximize phylogenetic information. The use of concatenated multilocus datasets with incomplete gene coverage is common practice in fungal systematics and has been shown to improve phylogenetic resolution despite missing data. Because ITS alone provided limited resolution within several lineages of *Xylaria*, the inclusion of LSU, *RPB2*, and *TUB2* substantially increased the number of informative characters and improved support for species delimitation and phylogenetic relationships. Sequence alignments were performed using Geneious Prime 2026.0.2 with the MAFFT plugin and algorithm G-INS-I and manually edited in the BioEdit Sequence Alignment Editor 7.0.5.3. Maximum likelihood analyses were performed on the concatenated four-locus dataset using IQ-TREE v2.4.0. The concatenated alignment of 78 sequences was initially partitioned by gene region. The best-fit nucleotide substitution model for each partition and the optimal partitioning scheme were simultaneously determined using ModelFinder (MFP + MERGE) [49], as implemented in IQ-TREE, under the Bayesian information criterion (BIC). This procedure allows merging of partitions with similar evolutionary patterns. Branch support was assessed using 1000 ultrafast bootstrap replicates (UFBoot2) and 1000 SH-aLRT replicates, with the -bnni option to minimize bootstrap overestimation. *Biscogniauxia arima* and *Biscogniauxia mediterranea* were selected as outgroup taxa and used to root the tree in FigTree v.1.4.3 [50]. Inclusion of all currently described species of *Xylaria* was beyond the scope of the present study and was not necessary to evaluate the taxonomic placement of the newly collected material.

## 3. Results

### 3.1. Phylogenetic Analysis

**Figure 2:**
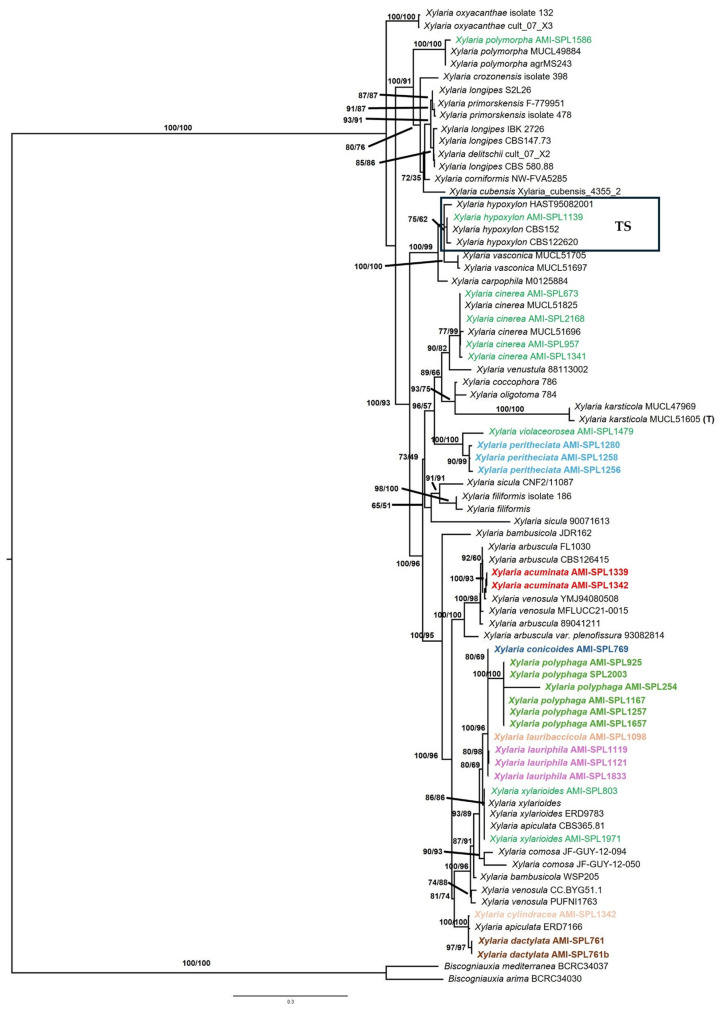
Maximum-likelihood phylogenetic tree of *Xylaria* inferred from a concatenated ITS–LSU–*TUB2*–*RPB2* dataset. The tree shown corresponds to the best-scoring ML topology (highest log-likelihood value). Branch support values are shown as SH-aLRT/ultrafast bootstrap percentages. Newly described species are highlighted in different colors, whereas newly generated sequences obtained in this study are indicated in light green. *Biscogniauxia arima* and *B. mediterranea* were used as outgroups. TS = type species of the genus *Xylaria.*

After trimming ambiguously aligned regions, the final combined dataset comprised 3527 characters, of which 1280 were parsimony informative. *Biscogniauxia arima* and *B. mediterranea* were selected as outgroups, and the tree was rooted accordingly. The best-scoring maximum likelihood tree inferred with IQ-TREE is shown in Figure 2.

In the resulting phylogram, the potential new taxa are distributed across several well-supported lineages that generally correspond to morphologically recognizable groups within *Xylaria*, although not without exceptions.

A strongly supported clade (SH-aLRT 100/BS 96) includes *Xylaria polyphaga*, *X. conicoides*, *X. lauribaccicola*, *X. lauriphila*, and *X. xylarioides*. Within this assemblage, *X. polyphaga* and *X. conicoides* form a basal subclade, whereas *X. lauribaccicola* occupies an intermediate position and *X. lauriphila* is closely allied to *X. xylarioides*, suggesting some internal structuring of the lineage, partly correlated with ecological differentiation.

Another well-supported lineage (SH-aLRT 100/BS 100) corresponds to the *X. apiculata* group, within which *X. cylindracea* and *X. dactylata* form a clearly delimited subclade (SH-aLRT 97/BS 97), characterized by distinctive stromatal features but differing markedly in fertile apex morphology.

In contrast, *X. acuminata* is firmly placed within the *X. arbuscula* sensu lato clade (SH-aLRT 100/BS 100) but clearly separated from the *X. venosula* lineage (SH-aLRT 100/BS 93), despite certain superficial similarities with members of the *X. apiculata* group, thus illustrating the limited phylogenetic value of some macromorphological characters when considered alone.

*Xylaria peritheciata* forms a distinct and well-supported lineage (SH-aLRT 90/BS 99), closely allied to *X. violaceorosea*, but clearly remote from the *X. sicula*–*X. filiformis* clade as well as from the *X. cinerea*–*X. oligotoma* assemblage.

Overall, the phylogeny supports the recognition of the newly described taxa as independent lineages, while highlighting the complexity of morphological evolution in *Xylaria* and the need for an integrative approach combining stromatal characters, micromorphology, ecology, and molecular data.

### 3.2. Taxonomy

***Xylaria acuminata* A. Mateos & De la Peña-Lastra, sp. nov.**

**MycoBank No. 862989**

**Figure 3:**
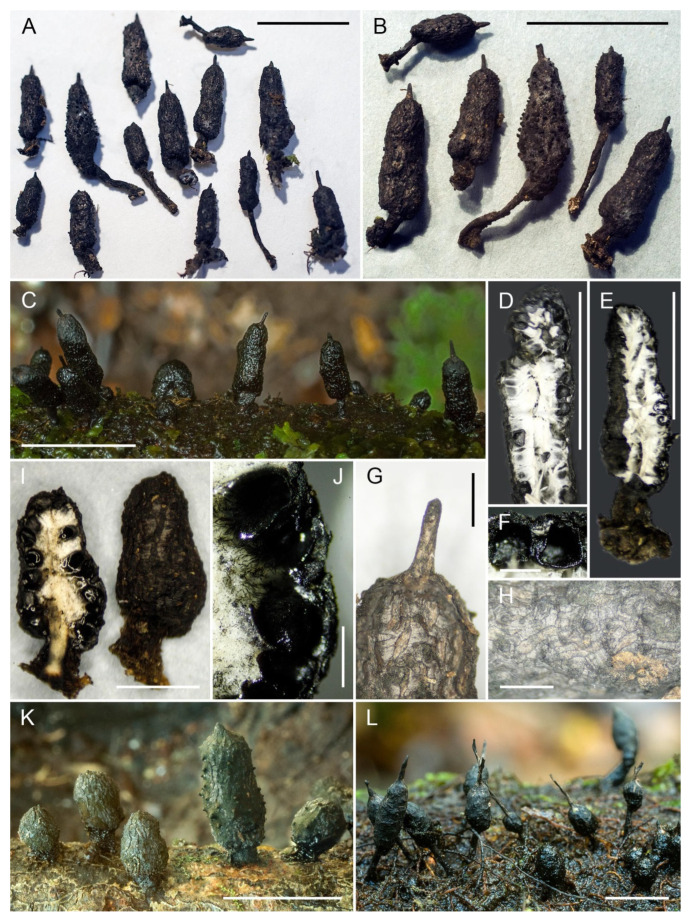
*Xylaria acuminata*. Macromorphology. (**A**–**H**) AMI-SPL1339 (Holotype), AMI-SPL1343, and AMI-SPL1344. (**A**,**B**) Morphological variability of the stromata. (**C**,**K**,**L**) Mature stromata with morphological variability on the host natural substrate *Laurus azorica* wood, in situ. (**D**,**E**) Stroma in vertical section showing perithecia fully immersed in a white fibrous internal tissue. (**F**,**J**) Perithecia vertical section fully immersed. (**I**) Outer and inner stroma in vertical section. (**G**,**H**) Mucronate cylindrical stroma showing silver–gray and light brown surface stripes with ostioles. Scale bars: (**A**–**C**) = 10 mm, (**D**,**E**,**I**,**K**,**L**) = 5 mm, (**G**,**H**) = 1 mm, and (**F**,**J**) = 0.5 mm.

**Figure 4:**
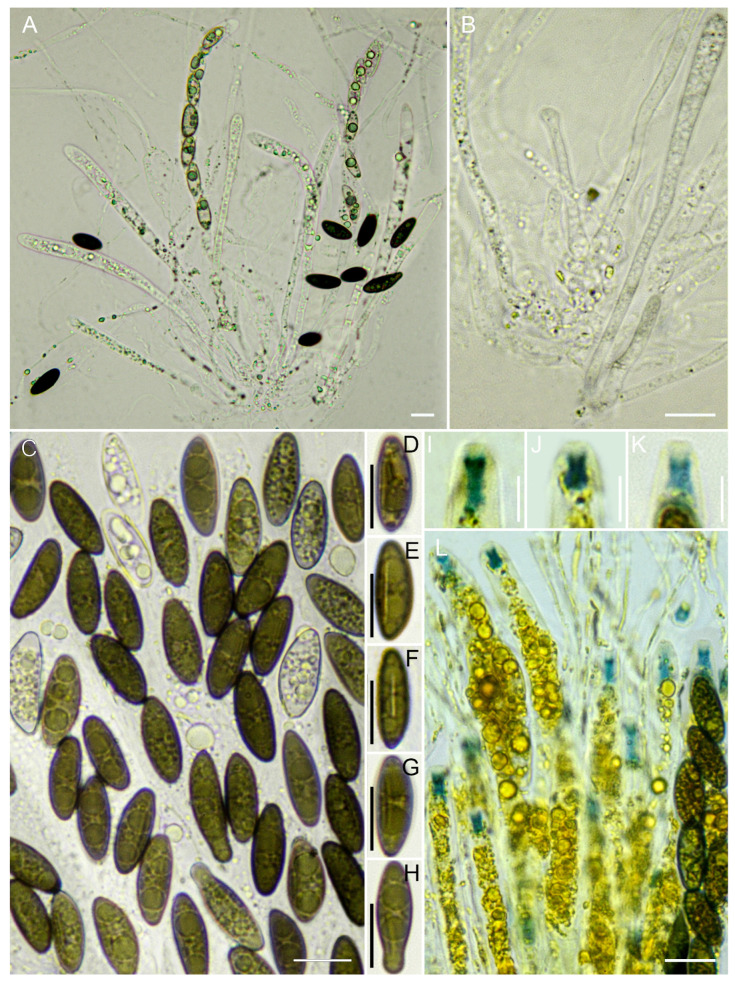
*Xylaria acuminata*. Micromorphology. (**A**–**L**) AMI-SPL1339 (Holotype). (**A**) Mature and immature asci with uniseriate ascospores, in water. (**B**) Immature asci with details of the base, in water. (**C**) Ascospores mature and immature, in water. (**D**–**H**) Ascospores in ventral view showing the germ slit, in water. (**I**–**K**) Ascal apical apparati in IKI-2 reagent. (**L**) Mature and immature asci, in IKI-2 reagent. Scale bars: (**A**–**H**,**L**) = 10 μm and (**I**–**K**) = 5 μm.

Figure 3 and Figure 4

**Etymology.** The specific epithet *acuminata* derives from the Latin *acuminatus*, meaning “tapering to a point” or “sharply pointed.” The name refers to the distinctly pointed apex of the stromata, which narrows gradually into an acute tip, often expressed as a mucronate or apiculate extension.

**Teleomorph. Stromata** generally densely gregarious, in large groups, sometimes solitary, simple, (8.5–)12.5–16(–18.5) mm total height, erect, subsessile to long-stipitate; fertile part 4–10 mm high × 2–3 mm broad, subglobose in juvenile stages but soon becoming subcylindrical to fusiform, terete, straight, terminating in short sterile mucronate–apiculate extensions 1–1.5 mm long, or much longer in other collections (4.5–5 mm), fragile, with irregular longitudinal strips or plaques, perithecial contours not exposed. Stromatal surface rough, grayish brown with black granulations, dark brown, becoming blackish with age, with occasional pinkish streaks at the apex; cortex carbonaceus, hard-textured, leathery, up to 110(–120) μm thick; interior white, homogeneous, fibrillose–spongy, somewhat hollow. **Stipe** sharply defined, 3–7 mm high × 0.4–0.6 mm diameter (diam.), terete, slightly swollen at the base (to 3 mm), subsurface black, leathery, densely covered with stiff black hairs, tomentose at the base. **Perithecia** generally immersed, spherical to subspherical, 600–700 μm high × 500–600 μm diam. **Ostioles** black, very conspicuous, and protruding at maturity, with conical to blunt papilla, up to 220 μm diam. at the base. **Asci** narrowly cylindrical, (100–)102.4–182.4(–200) μm total length, with a spore-bearing part 90–120 μm long × 6–7.1 μm wide, containing eight ascospores arranged uniseriately, base aporhynchous, apical apparatus tubular to urn-shaped, (3.1–)3.2–4.3(–6.4) × 1.9–2.2(–2.5) μm, euamyloid in Melzer’s reagent and Lugol’s solution. **Paraphyses** copious, filiform, thin-walled, equal to or longer than asci, 2–3 μm wide, slightly septate, filled with oily guttules, embedded in a gelatinous matrix, best observed in fresh material. **Ascospores** (13.9–)14.5–15.7–17.4(–19.3) × (5.2–)5.4–5.8–6.3(–6.7) µm, Q = (2.3–)2.4–2.7–3(–3.7), N = 50, Ve = 274 μm^3^, ellipsoid–inequilateral, with broadly rounded ends, something unusual with a slightly pointed tip, cellular appendage not observed, dark olive to medium brown, smooth, biguttulate, germ slit straight, extending 2/3–4/5 of the spore length on the less convex side. Aberrant ascospores with beak-like ends are occasionally observed.

**Habitat and Distribution.** Gregarious, growing in groups on wood of *Laurus azorica*. Known only from the Azores.

**Typus.** Portugal, Azores, Terceira, Angra do Heroísmo, Terra-Chã, Matela de Baixo, 38°41′59″ N, 27°15′31″ W, 470 m a.s.l., growing in groups on *Laurus azorica* wood, 16 January 2022, *leg.* A. Mateos & S. De la Peña, AMI-SPL1339.

**Additional material examined.** Portugal, Azores, Terceira, Angra do Heroísmo, Terra-Chã, Matela de Baixo, 38°41′59″ N, 27°15′31″ W, 470 m a.s.l., growing in groups on *Laurus azorica* wood, 16 January 2022, *leg.* A. Mateos & S. De la Peña, AMI-SPL1343; *ibid.*, AMI-SPL1344.

**Notes**. *Xylaria acuminata* is placed within the *Xylaria arbuscula* sensu lato clade, forming a distinct and moderately supported lineage (SH-aLRT 92/BS 60) within a well-supported group (SH-aLRT 100/BS 93) that includes *X. arbuscula* and *X. venosula*. This placement is consistent with its morphological affinities to members of the *X. arbuscula* complex.

*Xylaria acuminata* shares several features with members of the *X. arbuscula* complex, including small stroma, carbonaceous, cylindrical with non-exposed perithecial contours, a germ slit shorter than the spore length, and the absence of spore appendages; however, it has other clearly distinct features, such as never-branching stroma, a stromal surface with irregular, intermingled bands, and much larger spore measurements.

Several collections from France and Spain (Canary Islands) [9], Martinique [15], and Taiwan [17,51] have been published as *X. arbuscula* var. *plenofissura* and exhibit spores similar to or even larger than those observed here ((17.8–)18.2–19.9(–21.0) × (6.1–)6.2–7.3(–7.8) μm; Q = (2.5–)2.6–3.1(–3.5), Me = 19.0 × 6.8 μm) [8], but they differ in the germ slit, which occupies almost the entire length of the spore and is sigmoid rather than straight; furthermore, they have a transient cellular appendage on the immature spores.

*Xylaria venosula* appears phylogenetically close (USA, Hawaiian Islands, Ju & Hsieh 94080508 [52]), in agreement with the present phylogenetic results (SH-aLRT 88/BS 69), but represents a distinct species from *Xylaria acuminata*, both according to the original description and to subsequent interpretations, [53], who revised Spegazzini’s material and examined additional specimens collected in Tucumán (Argentina), where the original material was found. They concluded that it is a synonym of *Xylaria xylarioides*; in that taxon, stromata are morphologically distinct, ranging from subglobose to rarely conical, coated in strips, and bearing larger ascospores than those of *X. acuminata* (17–21 × 6.5–9 μm, Me = 18.3 × 7.7 μm vs. (13.9–)14.5–17.4(–19.3) × (5.2–)5.4–6.3(–6.7) μm, Me = 15.7 × 5.8 μm), and in the examination of the type specimen (17.5–21.5 × 7.5–9 µm), with a germ slit extending almost the entire spore length and acute ends. These differences are also maintained in Fournier et al. [10].

Although phylogenetically affiliated with the *X. arbuscula* complex, *X. acuminata* differs consistently from all currently recognized members of the group by its unbranched stromata, distinctive stromatal surface ornamentation, ascospore dimensions, and germ slit morphology. These stable morphological differences justify its recognition as a distinct species. The phylogenetic analysis is consistent with its placement within the *X. arbuscula* complex but is not used here as the primary criterion for species delimitation.

***Xylaria cinerea* J. Fourn. & M. Stadler, Mycol. Progr. 10(1): 36 (2011)**

**Figure 5:**
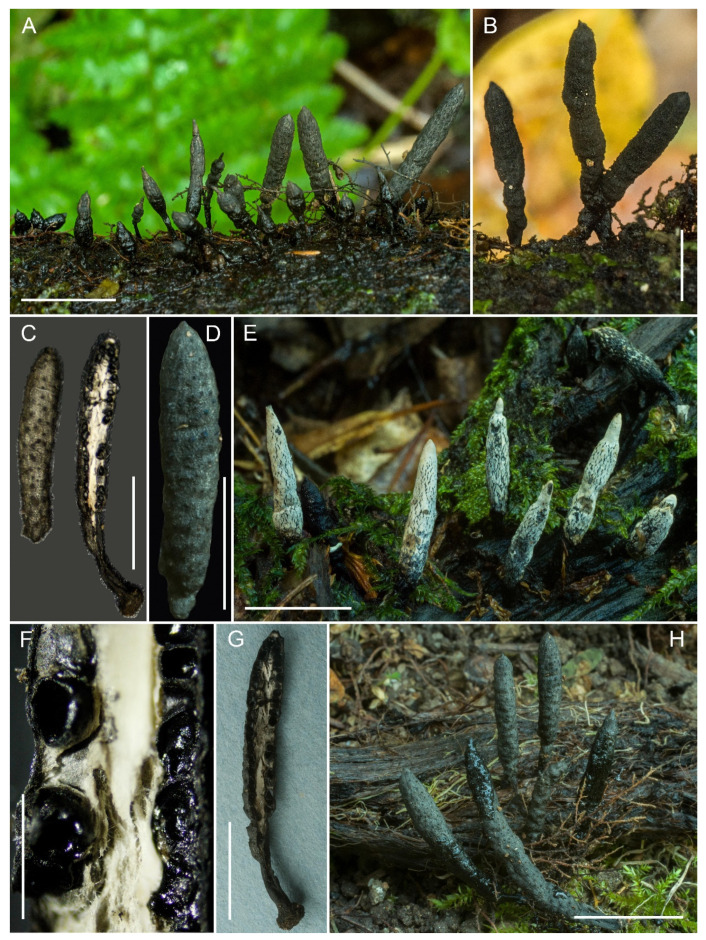
*Xylaria cinerea*. Macromorphology. (**A**–**D**,**F**–**H**) AMI-SP1341. (**E**) AMI-SPL773. (**A**,**B**,**H**) Mature teleomorphic stromata, on host surface of *Laurus azorica*, in situ. (**E**) Habit of conidial stromata (**H**), on host surface of *Laurus novocanariensis*, in situ. (**A**,**D**,**E**,**H**) Mature and conidial stroma showing a sterile apiculate apex. (**C**,**D**) Stromatal surface with ostioles, on a hairy stipe. (**C**,**F**,**G**) Stroma in vertical section showing perithecia fully immersed in a white fibrous internal tissue. Scale bars: (**A**,**E**,**H**) = 10 mm, (**B**–**D**,**G**) = 5 mm, and (**F**) = 1 mm.

**Figure 6:**
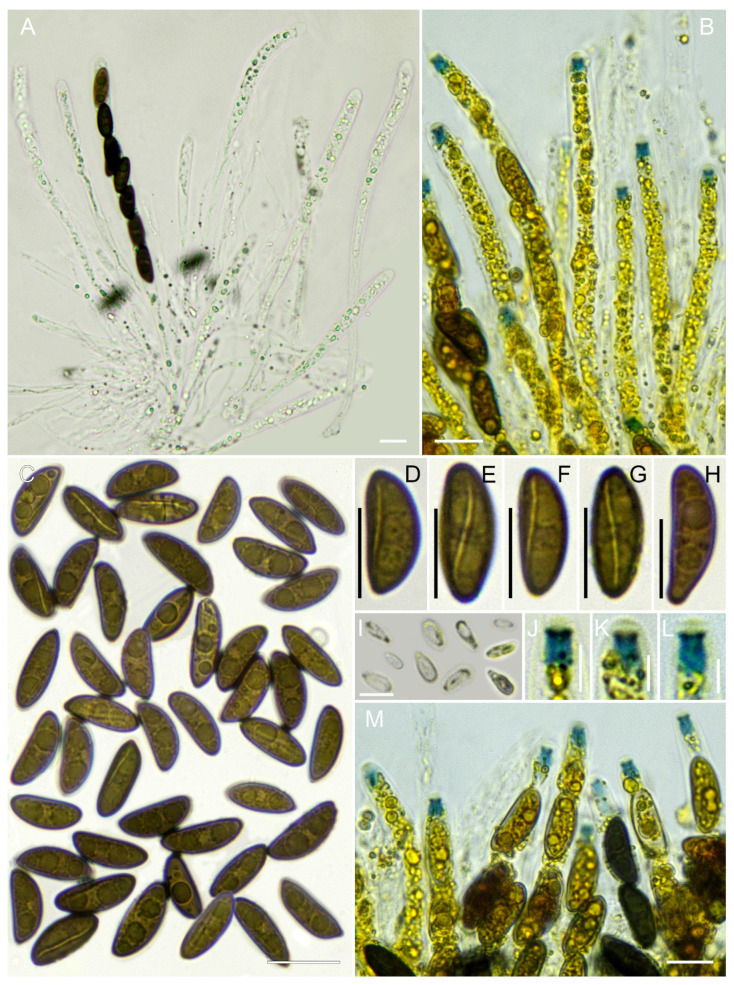
*Xylaria cinerea*. Micromorphology. (**A**–**H**,**J**–**M**) AMI-SPL1341. (**I**) AMI-SPL1340. (**A**) Mature and immature asci with uniseriate ascospores, in water. (**B**,**M**) Mature and immature asci, in IKI-2 reagent. (**D**–**G**) Ascospores showing the germ slit, in water. (**H**) Ascospores occasionally aberrant with beaked ends, in water. (**J**–**L**) Ascal apical apparati, in IKI-2 reagent. (**I**) Conidia clavate to pip-shaped, in water. Scale bars: (**A**–**H**,**M**) = 10 μm and (**I**–**L**) = 5 μm.

Figure 5 and Figure 6

**Teleomorph**. **Stromata** solitary but most often densely clustered, simple or very rarely branched, (16–)21–23(–25) mm total height, erect, stipitate; fertile portion 10–19 mm high × 1–2.5 mm broad, terete, subcylindrical to fusiform, straight, terminating in short sterile apex, 0.5–2 mm long, narrowly rounded or mucronate, fragile, generally flattened but sometimes nodulose, with longitudinal strips or plaques. Surface initially smooth, covered by a cream white to ochraceous fibrillose layer with longitudinal fissures, this layer tending to disappear; becoming silvery gray with black granulations, later blackish; cortex up to 50 μm thick, hard-textured, leathery; interior white, becoming darker at maturity, homogeneous, soft-spongy. **Stipe** sharply defined, 4–7(–12) mm high × 0.5–1.5 mm diam., terete, slightly swollen at the base (to 2 mm); surface black, leathery, tomentose. **Perithecia** generally immersed, sometimes slightly exposed, spherical to subglobose, 550–600 μm high × 400–500 μm diam. **Ostioles** black, scarcely papillate when young but evident at maturity, somewhat conical, located among the gray strips. **Asci** narrowly cylindrical, (110–)130–170(–190) μm total length, with a spore-bearing portion 60–100 μm long × 6–7(–9) μm wide, containing eight ascospores arranged uniseriately, sometimes slightly overlapping; base pleurorhynchous; apical apparatus tubular to urn-shaped, 2.3–2.7 × 3.5–4(–5) μm, euamyloid in Melzer’s reagent and Lugol’s solution. **Ascospores** (13–)13.5–14.6–15.7(–17.9) × (4.7–)4.9–5.4–5.9(–6.7) μm, Q = (2.2–)2.4–2.7–3.1(–3.4), N = 50, Ve = 228 μm^3^, ellipsoid–inequilateral, with broadly rounded ends, or with one acute end and the other rounded, rarely with a distinctly pointed end, cellular appendage not observed, brown, smooth, biguttulate, with a long axis parallel or slightly diagonal, germ slit straight or markedly sinuous, 2/3–4/5 the spore length, located on the less convex side. Aberrant ascospores with beak-shaped ends are observed with some frequency. **Conidia** fusiform–clavate, 4–5 × 2–2.5 μm, hyaline, smooth.

**Notes**. *Xylaria cinerea* is easily recognized in the juvenile stage by its whitish fibrillose pruina with longitudinal fissures. In the mature stage it is more ambiguous and should be identified based on the combination of black granules on a gray stromatal surface and relatively large ascospores with a germ slit often oblique or markedly sinuous [8]. Other European references include those cited in the works of Henrici [54,55] and Fournier et al. [9].

**Specimens examined**. **Madeira Islands.** Portugal, Madeira, São Vicente, Chão dos Louros, 32°45′37.1″ N, 17°00′58.5″ W, 820 m a.s.l., growing in groups on *Laurus novocanariensis* wood, 19 November 2021, *leg.* A. Mateos & S. De la Peña, AMI-SPL773; *ibid.*, AMI-SPL789. **Canary Islands.** Spain, Canary Islands, Santa Cruz de Tenerife, Monte del Agua, Parque Rural de Teno, 28°19′45.8″ N, 16°48′36.2″ W, 990 m a.s.l., growing in groups on *L. novocanariensis* wood, 3 December 2021, *leg.* A. Mateos & S. De la Peña, AMI-SPL1034. Santa Cruz de Tenerife, Monte de las Mercedes, Parque Rural de Anaga, 28°31′49.26″ N, 16°17′09.67″ W, 922 m a.s.l., growing in groups on *L. novocanariensis* wood, 2 December 2021, *leg.* A. Mateos & S. De la Peña, AMI-SPL914; *ibid.*, AMI-SPL957, AMI-SPL958. **Cortegada Islands.** Spain, Galicia, Pontevedra, Parque Nacional de las Islas Atlánticas de Galicia, Cortegada Island, 42°36′57.5″ N, 8°47′05.3″ W, 2 m a.s.l., growing in groups on *Laurus nobilis* wood, 23 March 2021, *leg.* A. Mateos & S. De la Peña, AMI-SPL673; *ibid.*, 24 February 2019, AMI-SPL2168; *ibid.*, 15 November 2017, AMI-SPL2059. **Azores Islands.** Portugal, Azores, Terceira, Angra do Heroísmo, Terra-Chã, Matela de Baixo, 38°41′59″ N, 27°15′31″ W, 470 m a.s.l., growing in groups on *Laurus azorica* wood, 16 January 2022, *leg.* A. Mateos & S. De la Peña, AMI-SPL1340; *ibid.*, AMI-SPL1341. **Parque Natural Los Alcornocales.** Spain, Cádiz, Los Barrios, Las Corzas, Parque Natural Los Alcornocales, 36°08′11.17″ N, 5°31′11.46″ W, 353 m a.s.l., growing in groups on *Laurus nobilis* wood, 17 December 2022, *A. Mateos*, *S. De la Peña & M. Plaza*, AMI-SPL1631. **Geoparque Villuercas-Ibores-Jara.** Spain, Extremadura, Cáceres, Geoparque Villuercas-Ibores-Jara, Alía, Lorera de la Trucha, Garganta de la Trucha, 39°32′50.57″ N, 5°14′55.08″ W, 640 m a.s.l., growing in groups on *Prunus lusitanica* wood, 16 January 2023, *leg.* A. Mateos, S. De la Peña & A. Gutierrez, AMI-SPL1739; *ibid.*, AMI-SPL1741.

***Xylaria conicoides* A. Mateos & De la Peña-Lastra, sp. nov.**

**MycoBank No. 862990**

**Figure 7:**
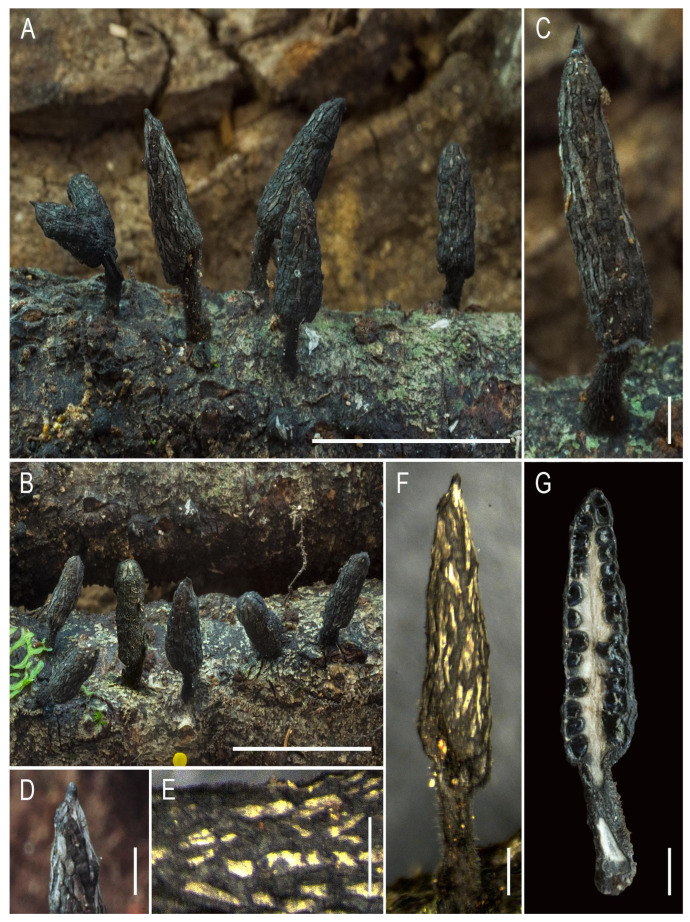
*Xylaria conicoides*. Macromorphology. (**A**–**G**) AMI-SPL769 (Holotype). (**A**,**B**) Mature stromata with morphological variability on the host natural substrate *Ocotea foetens* wood, in situ. (**C**–**F**) Mucronate cylindrical to fusiform stroma on showing silver–gray and tan superficial stripes. (**G**) Stroma in vertical section showing perithecia fully immersed in a white fibrous internal tissue, with a pinkish-gray inner core. Scale bars: (**A**,**B**) = 10 mm and (**C**–**F**) = 1 mm.

**Figure 8:**
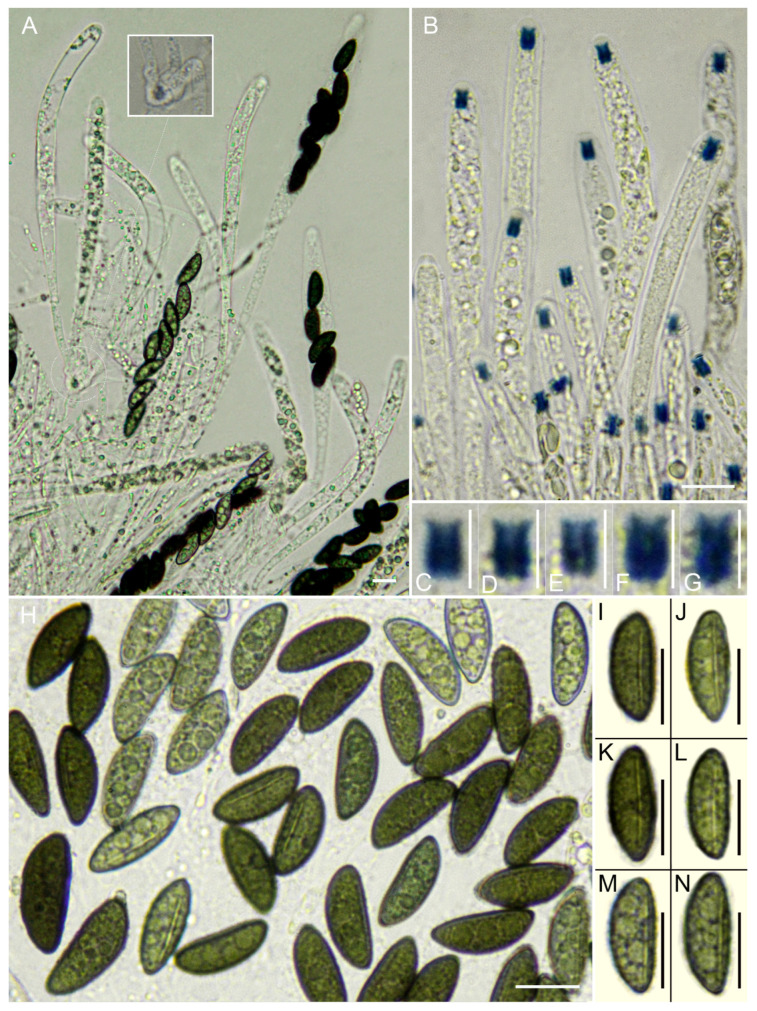
*Xylaria conicoides*. Micromorphology. (**A**–**N**) AMI-SPL769 (Holotype). (**A**) Mature and immature asci with uniseriate to biseriate ascospores with details of the base, in water. (**B**) Mature and immature asci, in IKI-2 reagent. (**C**–**G**) Ascal apical apparati in IKI-2 reagent. (**G**,**L**) Mature and immature ascospores in water. (**I**–**N**) Ascospores in ventral view showing the germ slit, in water. Scale bars: (**A**,**B**,**H**–**N**) = 10 μm and (**C**–**G**) = 5 μm.

Figure 7 and Figure 8

**Etymology.** The specific epithet *conicoides* derives from the Latin *conicus*, meaning “conical,” and the Greek suffix *oides* (-οειδής), meaning “resembling” or “having the form of.” The name refers to the conical shape of the stromata, which resemble those of related conical species within the genus.

**Teleomorph. Stromata** solitary but generally gregarious, 6–10(–11) mm in total height, erect, stipitate; fertile part 5–7 mm high × 2–2.5 mm broad, terete, conical, cylindrical to fusiform, straight, terminating in a short sterile mucronate apex, 0.5–1.2 mm long, fragile, with perithecial contours not exposed, bearing broad, irregular, longitudinal carbonaceous strips. Surface glabrous to slightly roughened, with gray–silvery bands alternating with dark toasted-reddish areas, becoming ochraceous to blackish with age, often with longitudinal fissures; subsurface a leathery cortex up to 60–80 μm thick, hard-textured, leathery; interior tissue white, becoming darker pinkish-gray at maturity, homogeneous, soft and spongy. **Stipe** sharply defined, (2–)3–4 mm high × 1–1.5 mm diam., terete, slightly swollen at the base (–2 mm), subsurface black, leathery, tomentose. **Perithecia** immersed, not exposed, spherical to subspherical, 400–600 μm high × 400–500 μm diam. **Ostioles** black, slightly prominent, fairly inconspicuous, situated among the gray strips. **Asci** subclavate when immature, cylindrical at maturity, (110–)144–180(–196) μm in total length, with a spore-bearing part 80–105 μm long × (6–)6.5–9(–12) μm wide, containing eight ascospores arranged uniseriately, sometimes slightly overlapping, base aporhynchous, apical apparatus urn-shaped, to slightly tubular, 3.4–3.9(–4.3) × 2–2.4(–2.7) μm, euamyloid in Melzer’s reagent and Lugol’s solution. **Paraphyses** abundant, filiform, hypha-like, hyaline, thin-walled, equal to or longer than the asci, 1.5–2.5 μm wide, septate, embedded in a gelatinous matrix, filled with large oily guttules, best observed in living material. **Ascospores** (14–)14.6–15.5–16.3(–16.8) × (4.7–)5.3–5.8–6.2(–6.5) μm, Q = (2.2–)2.4–2.7–2.9(–3.3), N = 50, Ve = 271 μm^3^, ellipsoid to inequilateral, with broadly rounded ends, or one end acute and the other rounded, cellular appendage not observed, dark olive to medium brown, smooth, biguttulate, with a long axis parallel or slightly diagonal, germ slit straight to slightly sinuous, extending over 4/5 to nearly the entire length of the spore on the less convex side.

**Habitat and Distribution.** Gregarious, growing in groups on wood of *Ocotea foetens*. Known only from Madeira.

**Typus.** Portugal, Madeira, São Vicente, Chão dos Louros, 32°45′37.1″ N, 17°00′58.5″ W, 820 m a.s.l., growing in groups on *Ocotea foetens* wood, 19 November 2021, *leg.* A. Mateos & S. De la Peña, AMI-SPL769.

**Notes**. *Xylaria conicoides* is recovered within the well-supported clade (SH-aLRT 100/BS 96) that includes *X. polyphaga*, *X. lauribaccicola*, *X. lauriphila*, and *X. xylarioides*. Although relationships among some members of this lineage remain weakly resolved, *X. conicoides* consistently occupies this phylogenetic group and is distinguished from the other taxa by a unique combination of morphological and ecological characters (Table S1).

From a morphological standpoint, *Xylaria conicoides* exhibits several characters typical of the *X. arbuscula* complex; however, it is readily distinguished from that group by its ascospore dimensions (Me = 12.6 × 5 μm in Fournier et al. [15]). It shows closer affinity to *X. arbuscula* var. *plenofissura* (Me = 15.1 × 6.3 μm in CLL 5216, Martinique, Fournier et al. [15], vs. Me = 15.5 × 5.8 μm in *X. conicoides*). The length of the germ slit in *X. conicoides* is intermediate between that reported for these taxa and, therefore, distinct from both. The macromorphological habitus is also more similar to *X. arbuscula* var. *plenofissura*, particularly in the presence of longitudinal strips on the stromatal surface, although in the present case these are broader. Another subtle difference from that variety is that the stromata are never branched nor apically furcate.

*X. conicoides* is further characterized by stromata with a distinctly conical to acutely tapering fertile apex, a feature that readily distinguishes it from closely related species within its clade. In contrast, *X. polyphaga* exhibits subcylindrical to narrowly fusiform stromata with a shorter and less distinctly conical mucronate apex.

In addition, *X. conicoides* differs from *X. polyphaga* by having smaller ascospores and a more restricted ecological amplitude, being primarily associated with woody substrates, whereas *X. polyphaga* occurs on a wide range of substrates, including fruits and wood (Table S1).

Within the same clade, *X. conicoides* differs from *X. lauriphila* and *X. lauribaccicola* in stromatal morphology and, based on the material currently available, does not show the strict association with Lauraceae substrates observed in those species. It also differs from *X. xylarioides* in having distinctly elongated, stipitate stromata rather than compact, subglobose to conical forms (Table S1).

***Xylaria cylindracea* A. Mateos & De la Peña-Lastra, sp. nov.**

**MycoBank No. 862992**

**Figure 9:**
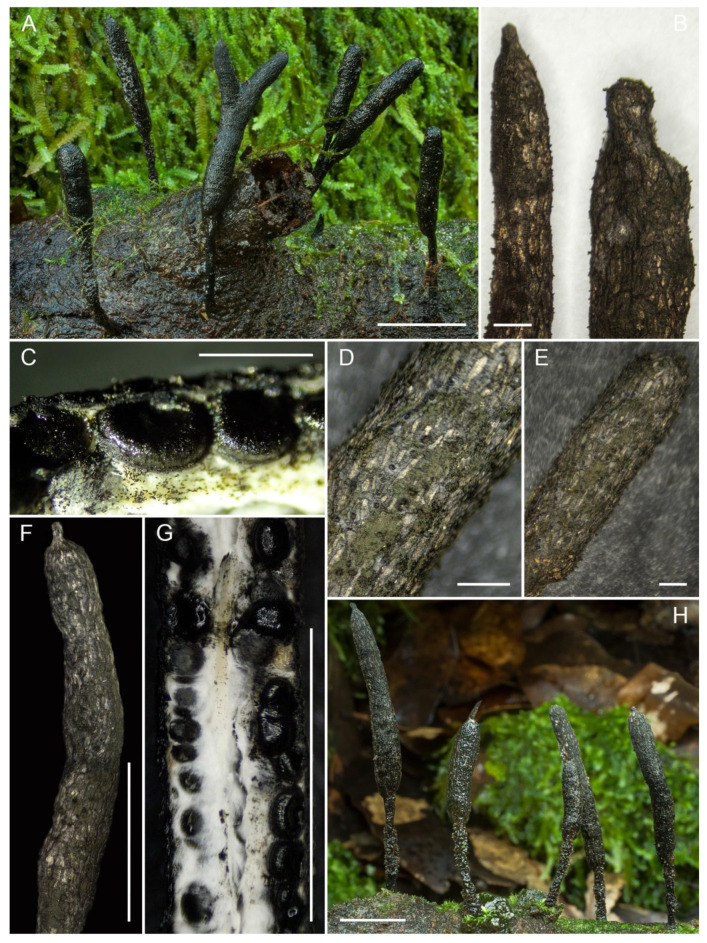
*Xylaria cylindracea*. Macromorphology. (**A**–**H**) AMI-SPL1342 (Holotype). (**A**,**H**) Morphological variability of the mature stromata on the host natural substrate *Laurus azorica* wood, in situ. (**B**,**F**) Cylindrical mucronate stroma showing silvery gray and cream-colored surface stripes. (**C**,**G**) Perithecia vertical section fully immersed in a white fibrous internal tissue. H: Stromatal surface and ostioles. Scale bars: (**A**,**F**,**H**) = 10 mm, (**G**) = 5 mm, (**B**,**D**,**E**) = 1 mm, and (**C**) = 0.5 mm.

**Figure 10:**
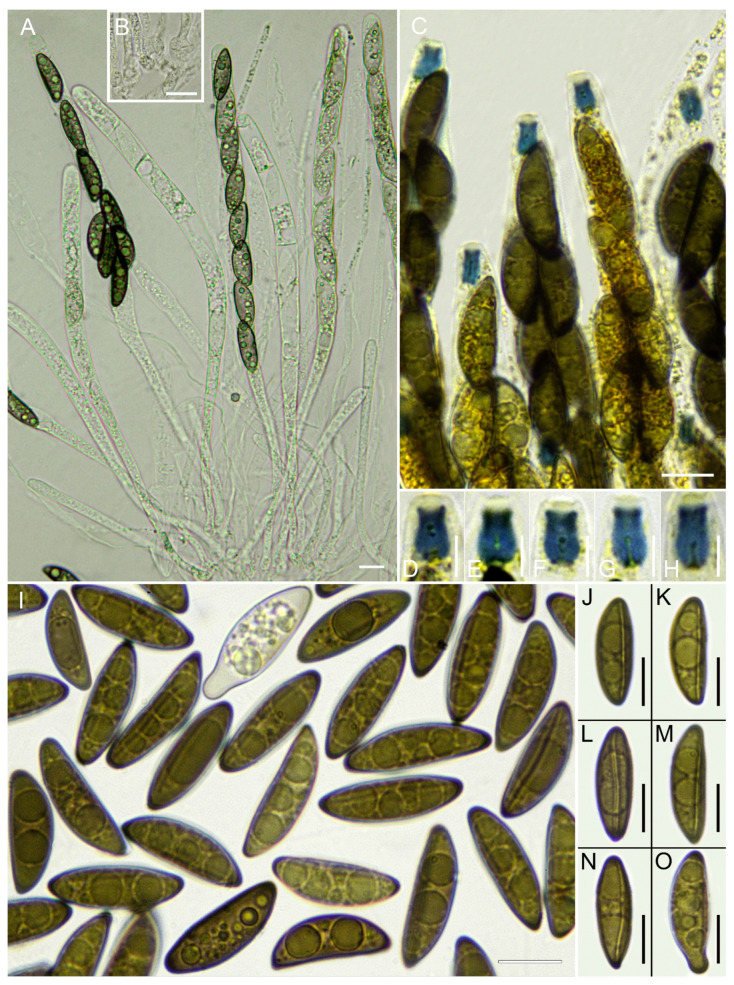
*Xylaria cylindracea*. Micromorphology. (**A**–**O**) AMI-SPL1342 (Holotype). (**A**) Mature and immature asci with uniseriate ascospores, in water. (**B**) Asci with details of the base, in water. (**C**) Mature and immature asci, in IKI-2 reagent. (**D**–**H**) Ascal apical apparati in IKI-2 reagent. (**I**) Mature and immature ascospores in water. (**J**–**N**) Ascospores in ventral view showing the germ slit, in water. (**O**) Ascospore occasionally aberrant with beaked end, in water. Scale bars: (**A**–**C**, **I**–**O**) = 10 μm and (**D**–**H**) = 5 μm.

Figure 9 and Figure 10

**Etymology.** The specific epithet *cylindracea* derives from the Latin *cylindraceus*, meaning “cylindrical” or “having the form of a cylinder,” ultimately from the Greek *kylindros* (κύλινδρος), meaning “cylinder” or “roller.” The name refers to the generally cylindrical shape of the stromata.

**Teleomorph. Stromata** gregarious, rarely solitary, simple, sometimes bifurcate in the fertile part, 33–45(–50) mm total height, erect, stipitate; fertile portion (10–)24–37 mm high × 3–4(–4.8) mm broad, terete, cylindrical, straight or slightly curved, terminating in a short sterile mucronate–apiculate apex, 0.5–1 mm long, fragile, with irregular longitudinal strips or plaques, perithecial contours not exposed. Surface rough, cracked, grayish, becoming blackish with age, with grayish and cream-colored strips; cortex leathery, 80–100 μm thick, hard-textured; interior white, homogeneous, fibrillose–spongy, becoming somewhat hollow at maturity. **Stipe** sharply defined, 12–23 mm high × 1.3–1.8 mm diam., terete, very widened at the base (to 3.8 mm), attached to the substrate, subsurface black, leathery, with dense, long black hairs, tending to disappear toward the upper part. **Perithecia** generally immersed, spherical or subglobose to ellipsoid, 400–800 μm wide × 500–850 μm high. **Ostioles** black, inconspicuous but numerous, raised-discoid, with a small, rounded papilla at the center. **Asci** cylindrical, (167–)177–263(–285) μm total length, with a spore-bearing portion 115–168 μm long × (6.6–)7–9.3(–12) μm wide, containing eight ascospores arranged uniseriately, base pleurorhynchous with croziers, apical apparatus urn-shaped, (5–)5.3–7.2(–7.5) × (3.1–)3.3–4.25(–4.3) μm, euamyloid in Melzer’s reagent and Lugol’s solution. **Paraphyses** copious, filiform, thin-walled, equal to or longer than asci, 2–4(–5) μm wide, somewhat embedded in a gelatinous matrix, filled with large oily guttules best seen in fresh material. **Ascospores** (19.5–)20.3–22.1–24.1(–24.8) × (6.4–)6.6–7.1–7.5(–8.7) μm, Q = (2.6–)2.8–3.1–3.5(–3.7), N = 50, Ve = 582 μm^3^, ellipsoid–inequilateral, with broadly rounded ends or slightly pinched on one side, some aberrant with one acute end, cellular appendage not observed, dark olivaceous to medium brown, smooth, generally biguttulate, but also monoguttulate or with three or more guttules, germ slit slightly curved to sigmoid, spore length, located on the less convex side.

**Habitat and Distribution.** Gregarious, growing in groups on wood of *Laurus azorica*. Known from the Azores (Terceira Island).

**Typus.** Portugal, Azores, Terceira, Angra do Heroísmo, Terra-Chã, Matela de Baixo, 38°41′59″ N, 27°15′31″ W, 470 m a.s.l., growing in groups on *Laurus azorica* wood, 16 January 2022, *leg.* A. Mateos & S. De la Peña, AMI-SPL1342.

**Notes**. *Xylaria cylindracea* is recovered within the *X. apiculata* lineage, forming part of a well-supported clade (SH-aLRT 100/BS 98) that includes *X. apiculata* and related taxa. Within this lineage, it is phylogenetically associated with *X. dactylata*, although it differs markedly from that species in stromatal morphology.

From a morphological standpoint, *Xylaria cylindracea* belongs to the *X. arbuscula* complex, more specifically to the *X. apiculata* clade, with which it shares strong macromorphological and micromorphological similarity. However, comparison with *X. apiculata* reveals several differences: the sterile apex is longer in *X. apiculata* (2–3 mm vs. 0.5–1 mm), the fertile head is slightly nodulose, and the surface bears grayish-black strips rather than cream-colored ones, as in *X. cylindracea*. The perithecia are somewhat larger (up to 1000–1200 μm diam.), the apical apparatus is cylindrical or tubular rather than urn-shaped and smaller, measuring (3.5–)4–5 μm high × 3–4(–5) μm wide vs. 5–7.5 × 3.1–4.3 μm, and the ascospores are larger (Me = 24 × 7.3 μm vs. 22.1 × 7.1 μm) [11,56].

Other species within this complex from which *Xylaria cylindracea* clearly differs are recovered in other clades (Figure 2) and include *X. arbuscula*, *X. arbuscula* var. *plenofissura*, *X. bambusicola*, *X. papillata*, *X. pseudoapiculata*, *X. schreuderiana*, *X. smilacicola*, *X. venosula*, and *X. xylarioides*, for which we refer to the references and detailed circumscriptions provided by Rubio [11].

In comparison with *X. dactylata*, *X. cylindracea* differs in having a simple cylindrical fertile part with a short mucronate apex, whereas *X. dactylata* exhibits an arborescent, branched apex with digitiform projections.

With respect to *X. polyphaga*, *X. lauribaccicola*, *X. acuminata*, *X. conicoides*, and *X. lauriphila*, *X. cylindracea* is readily distinguished by having ascospores of different shape and larger size than in all those taxa (see the descriptions and notes of these species in this paper, Table S1).

*Xylaria cylindracea* is characterized by a combination of cylindrical stromata, a short mucronate apex, cream-colored longitudinal surface striations, an urn-shaped apical apparatus, and ascospore dimensions that distinguish it from other currently recognized members of the *X. apiculata* complex.

***Xylaria dactylata* De la Peña-Lastra & A. Mateos, sp. nov.**

**MycoBank No. 863017**

Figure 11

**Etymology.** The specific epithet *dactylata* derives from the Greek *dactylus* (δάκτυλος), meaning “finger,” combined with the Latin suffix-*ata*, meaning “provided with.” The name refers to the characteristic digitiform branching of the stromatal apex, which resembles a cluster of finger-like projections.

**Teleomorph. Stromata** usually gregarious, rarely solitary, simple, 19–28 mm total height, erect, stipitate; fertile part 7–15 mm high × 1–1.9 mm broad, terete, cylindrical, subcylindrical or fusiform, straight, occasionally slightly flabelliform, with an arborescent branched apex bearing sterile tips 1–3 mm long, fragile. Himenial surface somewhat roughened, with dark grayish elongated vertical streaks and longitudinal fissures, poorly evident due to immaturity, and hairy tomentose owing to the presence of short, fine, hirsute hairs; perithecial contours not exposed; cortex up to 60–70 μm thick, hard-textured, leathery; internal entostroma white, becoming grayish-amber in the medulla, homogeneous, dense, firm, caseous. **Stipe** generally well defined, cylindrical, 12–15 mm long × 0.8–1 mm diam., terete, slightly widened at the base (−1.2 mm), subsurface concolorous with the remainder of the stroma, turning black with age, entirely covered with long, coarse hairs. Himenial hairs cylindrical to conical, 82–146 μm long × 4–6 μm diam., thick-walled, with base subequal or widened and occasionally bifurcate up to 10 μm broad, strongly septate, brown-pigmented. **Perithecia** immersed, not exposed, spherical to subglobose, lacking a well-defined wall, 300–400 μm diam., appearing as greenish-gray gelatinous structures. **Ostioles** not observed. **Asci** and **ascospores** not observed due to immaturity. **Anamorph stromata** present on the upper part of the teleomorphic stroma, forming several peaks (5–15 *u*), arborescent, subcylindrical, terete apex, 1–3 mm long × 0.2–0.3 mm broad, with rounded tips and covered by abundant whitish to cream pulverulent pruina. Conidiogenous layer arranged in a dense palisade, conidiogenous cells terminal, cylindrical with rounded to clavate apex, (25.6–)27.3–30.3(–32) × (1.8–)1.9–2.1(–2.2) µm, hyaline. **Conidia** produced holoblastically in sympodial succession, hyaline, smooth, pip-shaped, ellipsoid to lacrymoid, clavate to subcylindrical, sometimes curved, with an indistinct truncate scar basally, (2.4–)3.3–4.3–5.2(–5.4) × (1.3–)1.5–1.8–2(–2.2) µm, Q = (1.6–)1.9–2.4–2.9(–4.8), N = 50, Ve = 7 µm^3^. Conidia observed germinating in their natural habitat.

**Habitat and Distribution.** Gregarious, growing in groups on wood of *Laurus novocanariensis*. Known from the Madeira Island.

**Typus**. Portugal, Madeira, Parque Natural do Ribeiro Frio, 32°44′11.4″ N, 16°53′11.1″ W, 940 m a.s.l., growing in groups on *Laurus novocanariensis* wood, 18 November 2021, *leg.* A. Mateos & S. De la Peña, AMI-SPL761; *ibid.*, AMI-SPL761b.

**Notes**. *Xylaria dactylata*, as indicated above, is placed within the *Xylaria apiculata* lineage, in a well-supported subclade with *X. cylindracea* (SH-aLRT 98/BS 100), from which it is clearly distinguishable.

Morphologically, *X. dactylata* is readily distinguished by its characteristic arborescent stromatal apex bearing multiple digitiform projections, a feature unique within this lineage. This branching pattern gives the stromata a markedly distinctive appearance and constitutes the main diagnostic character of the species.

In comparison with *X. cylindracea*, *X. dactylata* differs in having a branched, digitiform apex rather than a simple cylindrical fertile part with a mucronate apex and longitudinally striate surface. From *X. apiculata*, *X. dactylata* can be separated by its well-developed, repeatedly branched apex, whereas *X. apiculata* typically exhibits a simple or only slightly apiculate sterile tip. Although morphologically comparable in some respects to species such as *X. acuminata*, *X. dactylata* differs in apex morphology, lacking the simple acuminate apex characteristic of that species and instead exhibiting a distinctly branched structure.

It should be noted that, in the specimens examined from our collection, although they are preserved in the conidial stage, they clearly indicate that the teleomorphic phase is also present and relatively well developed, albeit still immature. The stromata exhibit a general morphology typical of the genus *Xylaria*; however, the arborescent branching observed in the conidial stage shows some degree of similarity to that found in species such as *X. hypoxylon* [8,34,57] or *X. arbuscula* [8], although in those taxa the morphology of the apical branches differs markedly from that observed in the present species.

*Xylaria tentaculata* also shows certain similarities; however, its appendages may be considerably longer (up to 15 mm), uniform, straight, and acutely pointed, typically oriented upwards. In addition, its cultured conidia are larger and differ in shape, being subglobose, in contrast to those of *X. dactylata* [18,58,59].

The placement of *X. dactylata* within the *Xylaria comosa* group appears evident from a morphological perspective, as species within this complex are characterized by appendage-like branching structures in the anamorphic stage, as well as by the presence of hairs covering the stromatal surface. According to Læssøe [60], one of the most distinctive features of *X. comosa* sensu stricto is the presence of long and persistent secondary appendages, which have been largely overlooked in the literature and were described (briefly) by Batista and Peres [61]. Dennis [62] described them as “remnants of a hyaline appendix, rounded at each end.” Does the stroma have a more or less branched structure? These structures are absent in *Xylaria dactylata*. Furthermore, *X. comosa* produces somewhat larger conidia (5.3–6.6 × 1.5–2 µm) and has conidiogenous cells of different shape (ellipsoid to oblong) and smaller size.

*Xylaria comosoides* is similar to *X. comosa*, but differs in teleomorphic characters, as no data on the anamorph were available at the time of its description [60]. In species of this complex, there is usually a globose apex from which the arms emerge, a feature that is not apparent in our collection [60,62]. Rogers [63] proposed a classification based on the mode of anamorph production, placing in Section II those species that develop on specialized peg- or hair-like appendages, such as those of the *X. comosa* group. Within this complex, species such as *X. axifera*, *X. griseo-olivacea*, *X. melicearum*, and *X. guareae* differ from *X. dactylata* in the morphology of their protuberances, conidial characters, and ecological preferences [64].

The unusual and distinctive nature of these structures prompted a more detailed study of the material, including molecular sequencing, and phylogenetic analyses confirmed that no closely related sequences are currently available. Two collections of *X. comosa* deposited by J. Fournier in GenBank (MK546718, MF038919), although based on relatively short ITS sequences, still show clear differences from *Xylaria dactylata*.

We, therefore, describe this taxon as a new species, despite the teleomorphic stage not being fully characterized due to the immaturity of the available material, from which asci and ascospores could not be observed; nevertheless, the anamorphic state is well defined and provides sufficient diagnostic characters for its recognition.

***Xylaria hypoxylon* (L.) Grev., Fl. Edin.: 355 (1824)**

**Figure 12:**
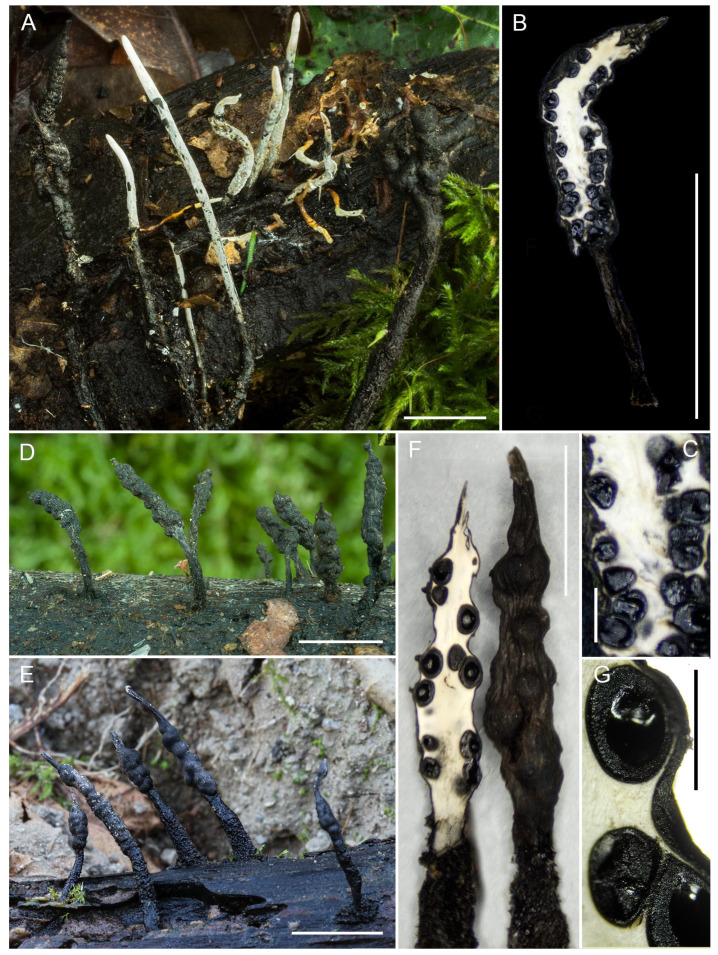
*Xylaria hypoxylon*. Macromorphology. (**A**–**C**) AMI-SPL1139. (**D**–**G**) AMI-SPL1819. (**A**) Stromata with morphological variability and teleomorph on the host natural substrate *Laurus novocanariensis* wood, in situ. (**B**) Stroma in vertical section showing perithecia. (**C**,**G**) Stroma in section showing perithecia and an interior white. (**D**,**E**) Stromata with morphological variability on the host natural substrate *Laurus nobilis* wood, in situ. (**F**) Stroma in vertical section showing perithecia fully immersed in a white fibrous internal tissue, and stromatal surface showing variously exposed perithecial contours and hemispherical ostioles. Scale bars: (**A**,**B**,**D**–**F**) = 10 mm and (**C**,**G**) = 1 mm.

**Figure 13:**
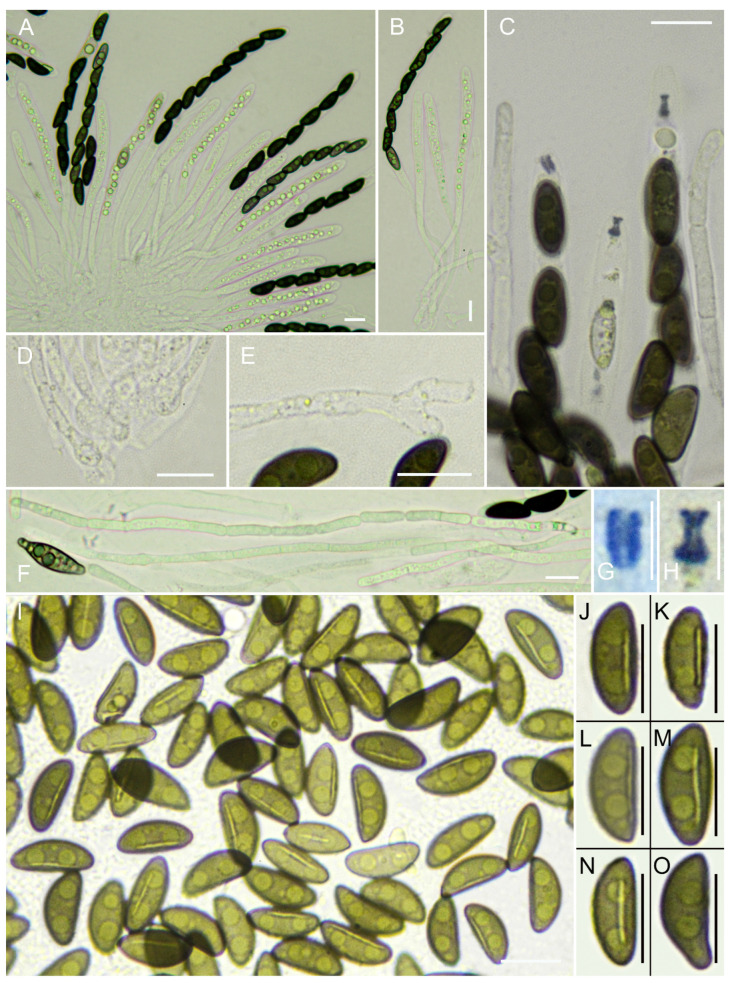
*Xylaria hypoxylon*. Micromorphology. (**A**–**O**) AMI-SPL1139. (**A**) Mature and immature asci with uniseriate ascospores, in water. (**B**) Mature and immature asci widened and rounded at the base, in water. (**C**) Mature and immature asci, in IKI-2 reagent. (**D**) Detail of the base of the asci. (**E**) Detail of the base of the paraphyses. (**F**) Septate moniliform paraphyses. (**G**,**H**) Ascal apical apparati in IKI-2 reagent. (**I**) Mature and immature ascospores in water. (**J**–**O**) Ascospores in ventral view showing the germ slit, in water. Scale bars: (**A**–**F**,**I**–**O**) = 10 μm and (**G**,**H**) = 5 μm.

Figure 12 and Figure 13

**Teleomorph. Stromata** variable, generally gregarious and sometimes solitary, simple, bifurcate or branched above the stipe into two or three fertile branches, occasionally further bifurcate, 26–80 mm total height, erect, stipitate; fertile portion 11–22 mm high × 2–2.5 mm broad, more or less fusiform to subcylindrical, terete to flattened, sometimes with an obtuse or conical mucronate sterile apex, frequently elongated, up to 5 mm long, perithecial contours more or less exposed. Surface striate and rough, forming narrow strips, whitish when young, becoming dark brown to grayish and later blackish; cortex up to 35(–65) μm thick, hard-textured, leathery, interior white to cream, homogeneous and solid, fibrous in places. **Stipe** very long and sometimes twisted, arising from roots, 20–45 mm high × 1–2 mm diam., terete, slightly swollen at the base (to 3 mm), surface black, rough-striate, hairy, more tomentose toward the base, leathery. **Perithecia** spherical to subglobose, 700–900 μm high × 500–700 μm diam. **Ostioles** prominent, raised discoid. **Anamorphic stromata** intermixed with mature teleomorphs, of similar length to the teleomorph though slenderer (1–1.5 mm thick), very filiform and straight, subcylindrical, vertical, with a conical, non-bifurcate apex, powdery with conidia in mass, anamorphic layer white, gradually disappearing, base attached to the substrate. **Asci** narrowly cylindrical, (150–)170–185(–210) μm total length, with a spore-bearing portion (80–)85–93(–100) μm long × (6–)6.5–7(–8.1) μm wide, containing eight ascospores arranged uniseriately, apical apparatus tubular to somewhat bell-shaped, 2.5–2.8 × 1.5–1.8 μm, euamyloid in Melzer’s reagent and Lugol’s solution. **Paraphyses** copious, filiform, thin-walled, sometimes bifurcate at the base, equal to or longer than the asci, (1.5–)2–3(–3.5) μm wide, distinctly septate, moniliform, filled with coarse oily guttules, embedded in a gelatinous matrix. **Ascospores** (10.7–)11.1–12.4–13.4(–14.8) × (4.1–)4.4–4.9–5.3(–5.7) μm, Q = (2.1–)2.3–2.5–2.8(–3.5), N = 50, Ve = 155 μm^3^, ellipsoid–inequilateral, with rounded ends, sometimes slightly narrowed at one end, cellular appendage not observed, straight or slightly curved, germ slit shorter than the spore length (1/2–4/5 of spore length), located on the less convex side.

**Notes**. This is a widely distributed and frequently encountered species that develops teleomorphs simultaneously with anamorphs. It is characterized by generally branched and long-mucronate stromata, the stromatal surface is striate, with perithecial contours more or less evident, and a long stipe that is rough and hairy at the base. The species was delimited by Peršoh et al. [34] and typified by Stadler et al. [57] and Fournier [8].

The ascospore size range renders it similar to several species with which it may be confused, including *Xylaria arbuscula*, *Xylaria karsticola*, and *Xylaria vasconica*. It differs from *X. arbuscula* in having stromata short-cylindrical to slightly fusiform, with a surface bearing large horny strips or plaques rather than being striate, a leathery texture, papillate ostioles, slightly larger ascospores (12.7–15.5 × 5–5.5 μm), and a slightly urn-shaped apical apparatus [9].

*X. karsticola* differs mainly in having a markedly nodulose and deeply wrinkled stromatal surface, ascospores with acute or nearly pinched ends, and longer germ slits; moreover, its anamorph is produced in late spring to early summer rather than in autumn–winter. *X. vasconica* is the most similar species, both in culture and in its anamorph and phylogeny. It differs from *X. hypoxylon* in consistently producing long cylindrical stromata growing in dense groups, which are neither branched nor flattened with bifurcate apices, and in having a strongly nodulose surface [9].

**Specimens examined. Canary Islands.** Spain, Canary Islands, Santa Cruz de Tenerife, La Gomera, Parque Nacional de Garajonay, 28°06′37.3″ N, 17°14′54.5″ W, 1470 m a.s.l., growing in groups on *Laurus novocanariensis* wood, 5 December 2021, *leg.* A. Mateos, S. De la Peña & D. Chavez, AMI-SPL1127; *ibid.*, AMI-SPL1139. Spain, Canary Islands, Santa Cruz de Tenerife, Monte del Agua, Parque Rural de Teno, 28°19′45.8″ N, 16°48′36.2″ W, 990 m a.s.l., growing in groups on *L. novocanariensis* wood, 3 December 2021, *leg.* A. Mateos & S. De la Peña, AMI-SPL990. **Parque Natural Los Alcornocales.** Spain, Cádiz, Los Barrios, Las Corzas, Parque Natural Los Alcornocales, 36°08′11.17″ N 5°31′11.46″ W, 353 m a.s.l., growing in groups on *Laurus nobilis* wood, 17 December 2022, *A. Mateos*, *S. De la Peña & M. Plaza*, AMI-SPL1639. **Geoparque Villuercas-Ibores-Jara.** Spain, Extremadura, Cáceres, Geoparque Villuercas-Ibores-Jara, Villar del Pedroso, Lorera del Mesto, Garganta del Mesto, Valle del Hospital del Obispo, 39°35′24″ N, 5°19′35″ W, 770 m a.s.l., growing in groups on *Prunus lusitanica* wood, 17 January 2023, *leg.* A. Mateos, S. De la Peña & A. Gutierrez, AMI-SPL1791. **Parque Nacional Peneda-Gerês.** Portugal, Braga, Terras de Bouro, Freitas, Parque Nacional Peneda-Gerês, 41°42′15.4″ N, 8°13′01.8″ W, 400 m a.s.l., growing in groups on *L. nobilis* wood, 16 February 2023, *leg.* A. Mateos & S. De la Peña, AMI-SPL1819; *ibid.*, AMI-SPL1824. **Madeira Islands.** Portugal, Madeira, Parque Natural do Ribeiro Frio, 32°44′11.4″ N 16°53′11.1″ W, 940 m a.s.l., growing in groups on *Laurus novocanariensis* wood, 18 November 2021, *leg.* A. Mateos & S. De la Peña, AMI-SPL733. Portugal, Madeira, Ribeira da Janela, Fanal, 32°48′55.9″ N 17°08′54.2″ W, 1100 m a.s.l., growing in groups on *L. novocanariensis* wood, 20 November 2021, *leg.* A. Mateos & S. De la Peña, AMI-SPL908.

***Xylaria lauribaccicola*** **De la Peña-Lastra & A. Mateos, sp. nov.**

**MycoBank No. 862988**

**Figure 14:**
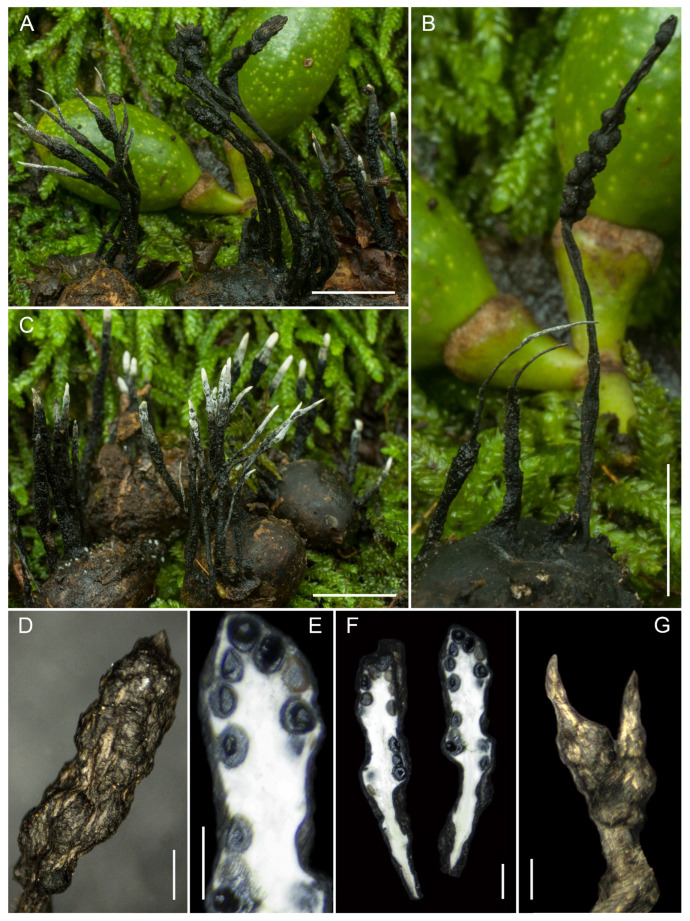
*Xylaria lauribaccicola*. Macromorphology. (**A**–**G**) AMI-SPL1098 (Holotype). (**A**,**B**) Mature stromata associated with asexual structures on the surface of *Laurus* berries, in situ. (**D**) Mucronate subcylindrical stroma. (**C**) Habit of conidial stromata on host surface, in situ. (**E**,**F**) Stroma in vertical section showing perithecia fully immersed in a white fibrous internal tissue, with a cream-pink inner core. (**G**) Long, sharp apiculi. Scale bars: (**A**–**C**) = 10 mm and (**D**–**G**) = 1 mm.

**Figure 15:**
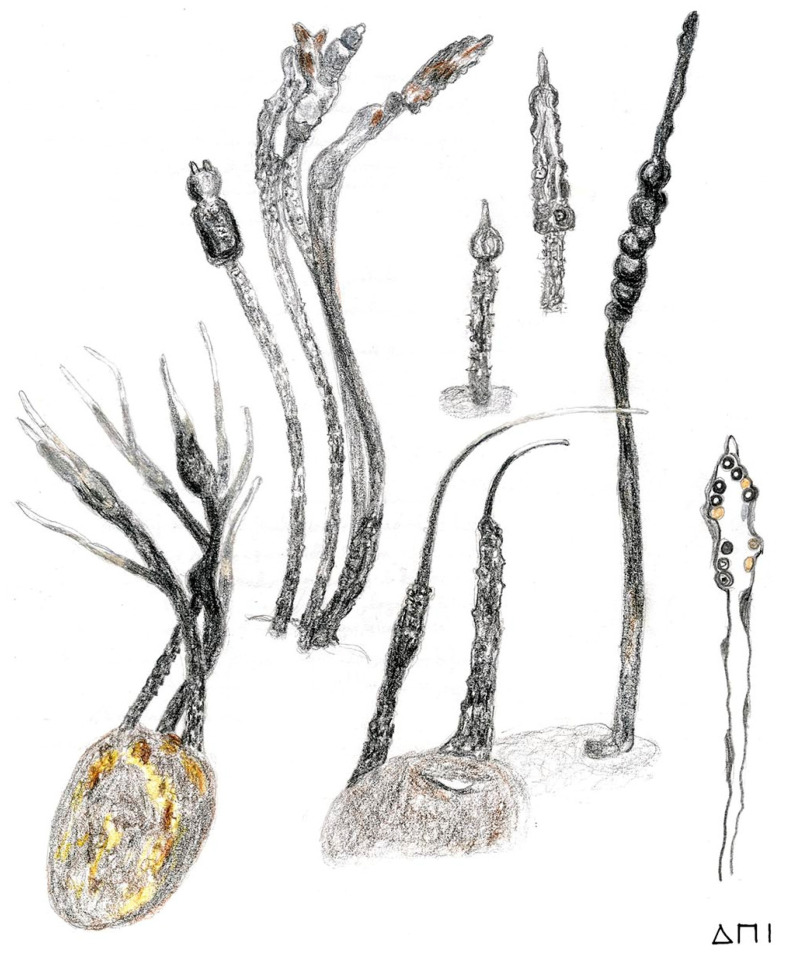
*Xylaria lauribaccicola*. Drawing by A. Mateos (AMI) of the holotype collection, AMI-SPL1098.

**Figure 16:**
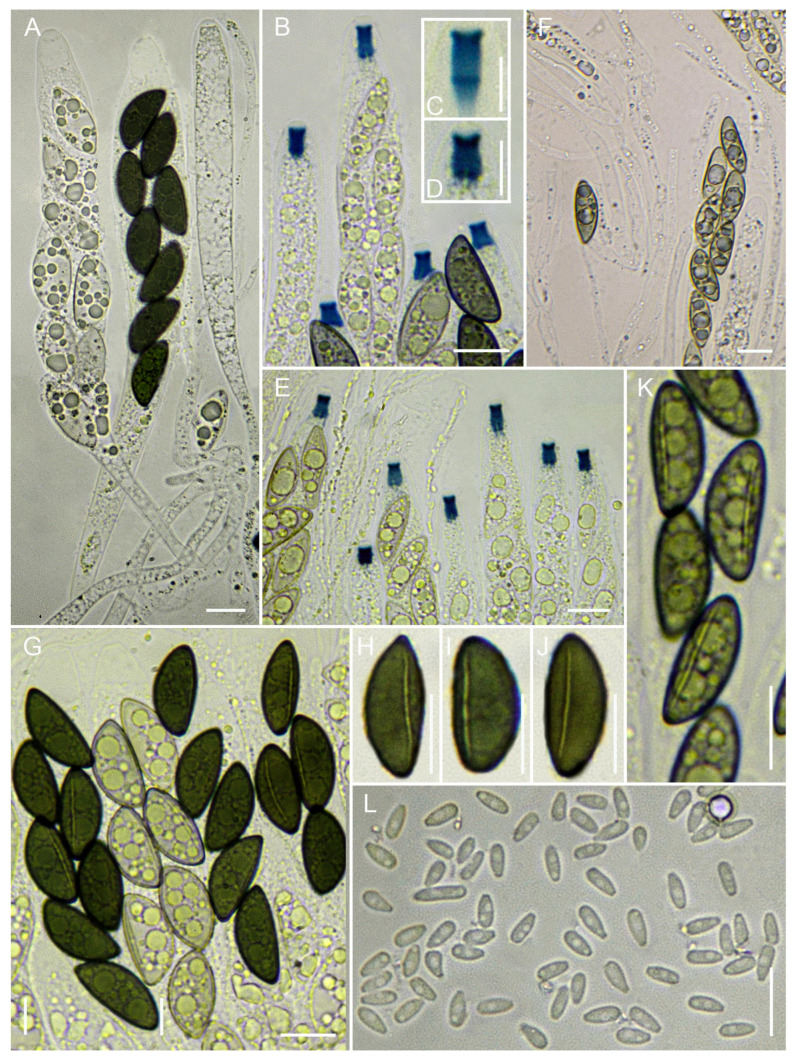
*Xylaria lauribaccicola*. Micromorphology. (**A**–**L**) AMI-SPL1098 (Holotype). (**A**) Mature and immature asci with uniseriate to biseriate ascospores, with details of the base, in water. (**B**,**E**) Mature and immature asci, in IKI-2 reagent. (**C**,**D**) Ascal apical apparati of immature asci, in IKI-2 reagent. (**F**) Paraphyses containing refractive guttules, in Congo red. (**G**) Mature and immature ascospores, in water. (**H**–**J**) Ascospores in ventral view showing the germ slit, in IKI-2 reagent. (**K**) Ascospores showing the germ slit, in water. (**L**) Conidia, in water. Scale bars: (**A**,**B**,**E**–**L**) = 10 μm and (**C**,**D**) = 5 μm.

Figure 14, Figure 15 and Figure 16

**Etymology.** The specific epithet *lauribaccicola* derives from the Latin *lauri*, the genitive of *Laurus*, bacca, meaning “berry” or “fruit,” and the suffix -*cola*, meaning “dweller” or “inhabitant.” The name refers to the ecological specialization of the species, which grows on the berries of *Laurus*, particularly those of *Laurus novocanariensis*.

**Teleomorph. Stromata** very variable, solitary but generally grouped in a subcespitose or gregarious manner, simple or more rarely furcate, total length 30–46 mm, erect and stipitate; fertile part 6–8 mm high × 1.2–2(–2.5) mm broad, terete, subcylindrical or fusiform, contorted or twisted, sometimes divided in two longitudinal lobes or constricted in places, with perithecia separated into two portions; apex sterile, mucronate, short, conical to obtuse, sometimes bearing a long, sharp apiculus, rarely double, fragile, 1–2 mm long. Stromatal surface gray, brown to blackish, becoming black with age, nodulose with perithecial contours slightly to moderately exposed when young, but perithecia becoming strongly exposed at maturity; surface rugose, composed of a dark gray outer layer divided into elongate, striate strips delimiting light brown fissures; subsurface coriaceous, ca. 10 μm thick, leathery, with an internal core white to pinkish cream, homogeneous, solid, with a cheesy to fibrous–spongy texture. **Stipe** well defined, very long and sinuous, (8–)20–29(–32) mm high × 0.8–1.2 mm diam., sometimes divided near the fertile part and sometimes near the base, generally cylindrical, subuniform, slightly widened at the base, blackish, densely covered with short hairs; stromata lacking pigments extractable in KOH. **Perithecia** subglobose, with or without exposed contours, sometimes in contact, 350–500(–600) μm high × 250–450 μm diam., brownish-yellowish when young, becoming black, surrounded by white-textured tissue. **Ostioles** black, with blunt papillate, 250 μm diam. at the base, inconspicuous. **Anamorphic stromata**, in the natural substrate, somewhat more filiform and twisted, with apices sometimes bifurcate, bearing conidia white in mass, total length 25–40 mm, the future fertile part 4–7 mm × (1–)1.5–1.7 mm, and stipe up to 1.2 mm diam., densely hairy. **Asci** cylindrical to fusiform, (100–)130–165(–180) μm in total length, with a spore-bearing part 80–100(–110) μm long × (6.5–)9.5–17 μm wide, containing eight oblique ascospores arranged uniseriately, biseriate in places, base aporhynchous, without clamp connection; apical apparatus tubular, slightly flared at the apex, 4.9–6.3 μm high × 2.5–3.3 μm broad, euamyloid in Melzer’s reagent and in Lugol’s solution. **Paraphyses** copious, filiform, hypha-like, hyaline, thin-walled, equal to or longer than the asci, 1.5(2)–4 μm wide, septate, embedded in a gelatinous matrix, filled with large oily guttules, observed in living material. **Ascospores** (15.8–)16.7–18.1–19.5(–21) × (6.5–)7.1–7.7–8.5(–9.5) µm, Q = (1.9–)2.1–2.6(–2.9), N = 50, Me = 18.1 × 7.7 µm, Qe = 2.4, Ve = 560 µm^3^, ellipsoidal–inequilateral, with both ends narrowly rounded to acute, dark olive to medium brown, smooth, generally biguttulate though sometimes with additional guttules, with a very conspicuous, straight, rarely slightly sinuous germ slit, extending about 4/5 of the spore length on the flattened ventral side, without visible sheath or appendages. **Conidia** 4.1–8 × 2.1–2.7 μm, clavate to pip-shaped, hyaline, smooth.

**Habitat and Distribution.** Gregarious, growing on the fruits of *Laurus novocanariensis*. Known only from La Gomera (Parque Nacional de Garajonay).

**Typus.** Spain, Canary Islands, Santa Cruz de Tenerife, La Gomera, Parque Nacional de Garajonay, 28°06′37.3″ N, 17°14′54.5″ W, 1470 m a.s.l., growing gregariously on the fruits of *Laurus novocanariensis*, 5 December 2021, *leg.* A. Mateos, S. De la Peña & D. Chavez, AMI-SPL1098.

**Additional material examined.** Spain, Canary Islands, Santa Cruz de Tenerife, La Gomera, Parque Nacional de Garajonay, 28°06′37.3″ N, 17°14′54.5″ W, 1470 m a.s.l., growing gregariously on the fruits of *L. novocanariensis*, 5 December 2021, *leg.* A. Mateos, S. De la Peña & D. Chavez, AMI-SPL1102.

**Notes**. *Xylaria lauribaccicola* is recovered within the well-supported clade (SH-aLRT 100/BS 96) that includes *X. polyphaga*, *X. conicoides*, *X. lauriphila*, *X. apiculata*, and *X. xylarioides*. This placement indicates its affinity with members of this group, although the phylogenetic analysis is not used here as the primary criterion for species delimitation.

*X. lauribaccicola* is distinguished by its occurrence on Lauraceae fruits, a feature that readily separates it from most species within this clade. In contrast, *X. polyphaga* exhibits a broad ecological amplitude across a wide range of substrates (Table S1).

The host plant on which our collections grow, the berries of *L. novocanariensis*, combined with their variable morphology, can present certain identification difficulties at first glance, particularly because the stipe can be very long (up to 32 mm in the type specimen) and because the perithecial contours are poorly evident in young specimens, becoming strongly exposed at full maturity; nevertheless, the presence of characteristic grayish strips on the surface of the fertile portion, together with the distinctly villose stipe and the diagnostic microscopic characters, allows reliable identification of this species.

It differs from *X. lauriphila*, which is also associated with Lauraceae, by its strict association with fruits rather than woody substrates, indicating a different ecological niche within the same host family. Additionally, *X. lauribaccicola* can be separated from *X. conicoides* and *X. xylarioides* by its ecological specialization and differences in stromatal structure and ascospore morphology (Table S1).

*Xylaria arbuscula* occupies a different host and shows a superficially similar morphology, but it is readily distinguished by its ascospores, which have non-acute ends, are markedly smaller, and possess a shorter germ slit [8,15]. *Xylaria polyphaga*, which occurs on a similar substrate and shares some morphological traits, differs in having ascospores with more rounded ends, slightly smaller dimensions ((10.9–)11.6–13.8–17.3(–18.5) × (4.7–)5–5.8–6.8(–7.2) µm), and a shorter germ slit (see the description in this paper).

Within this lineage, *X. lauribaccicola* is distinguished by its association with Lauraceae fruits, together with its stromatal morphology and ascospore characters, which differ from those observed in related species.

*Xylaria lauribaccicola* is recognized by a combination of ecological, macromorphological, and micromorphological characters, including its occurrence on Lauraceae fruits, characteristic stromatal features, and ascospore morphology. These characters allow its distinction from other currently recognized species within this group (Table S1).

***Xylaria lauriphila* De la Peña-Lastra & A. Mateos, sp. nov.**

**MycoBank No. 862991**

**Figure 17:**
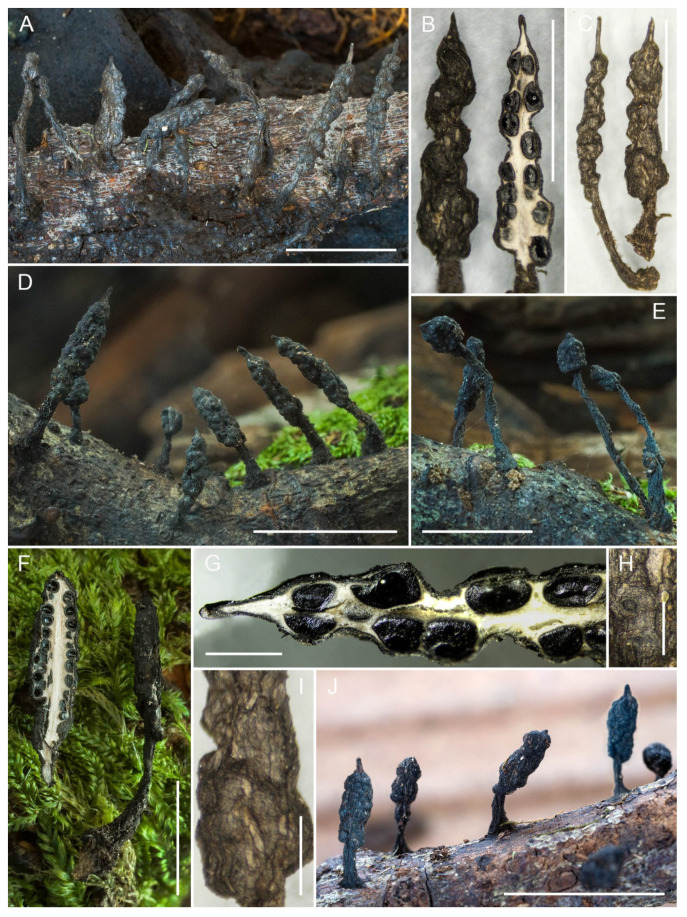
*Xylaria lauriphila*. Macromorphology. (**A**–**C**,**G**,**I**) AMI-SPL1833 (Holotype). (**D**–**F**) AMI-SPL1121. (**H**,**J**) AMI-SPL1119. (**A**,**D**,**E**,**J**) Morphological variability of the mature stromata on the host natural substrate *Laurus novocanariensis* wood, in situ. (**B**,**F**) Stroma exterior and interior in vertical section. (**C**) Mucronate cylindrical stroma showing silver–gray and tan superficial stripes. (**G**) Perithecia vertical section fully immersed in a white fibrous internal tissue. (**H**) Stromatal surface and ostioles. (**I**) Close-up of stroma showing silver–gray and tan superficial stripes. Scale bars: (**A**,**D**,**E**,**J**) = 10 mm, (**B**,**C**,**F**) = 5 mm, (**G**,**I**) = 1 mm, and (**H**) = 0.5 mm.

**Figure 18:**
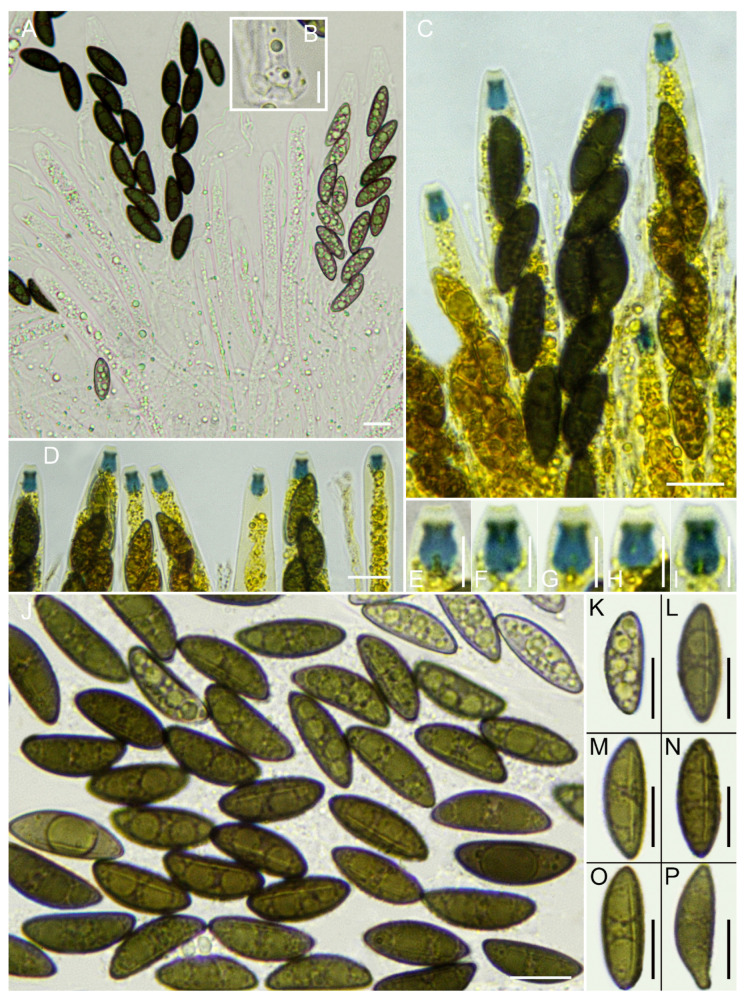
*Xylaria lauriphila*. Micromorphology. (**A**–**P**) AMI-SPL1833 (Holotype). (**A**) Mature and immature asci with uniseriate to biseriate ascospores, in water. (**B**) Asci with details of the base, in water. (**C**,**D**) Mature and immature asci, in IKI-2 reagent. (**E**–**I**) Ascal apical apparati in IKI-2 reagent. (**J**) Mature and immature ascospores in water. (**K**–**O**) Ascospores in ventral view showing the germ slit, in water. (**P**) Ascospore occasionally aberrant with beaked end, in water. Scale bars: (**A**–**D**,**J**–**P**) = 10 μm and (**E**–**I**) = 5 μm.

Figure 17 and Figure 18

**Etymology.** The specific epithet *lauriphila* derives from the Latin *laurus*, meaning “laurel,” and the Greek suffix -*philos* (φίλος), meaning “loving” or “having affinity for.” The name refers to the ecological association of the species with trees of the genus *Laurus*, on whose substrates the fungus is typically found.

**Teleomorph. Stromata** generally gregarious, rarely solitary, simple, 10.5–14(–15) mm total height, erect, short to long stipitate; fertile portion (3–)6.5–7 mm high × 1.5–1.8(–2) mm broad, terete, subcylindrical to fusiform, straight, terminating in medium to long sterile mucronate–apiculate apex, 1.2–1.8 mm long, fragile, with irregular longitudinal strips or plaques, perithecial contours slightly to distinctly exposed. Surface rough, grayish-brown to dark brown, becoming blackish with age, with pinkish or brownish strips; cortex leathery 44–80 μm thick, hard-textured; interior white to cream, yellowish in the medulla, homogeneous, fibrillose–spongy, somewhat hollow. **Stipe** sharply defined, 3–5 mm high × 0.4–0.6 mm diam., terete, very widened at the base (to 3 mm), attached to the substrate, subsurface black, leathery, with dense black stiff hairs, tomentose at base. **Perithecia** generally immersed, ovoid to ellipsoid, 350–600 μm wide × 600–800 μm high. **Ostioles** black, inconspicuous, becoming prominent at maturity, with rounded papillae up to 100 μm diam. at the base. **Asci** narrowly cylindrical, (107.3–)133.1–175.7(–187) μm total length, with a spore-bearing portion 80–112 μm long × (5.4–)6.6–10.9(–12.2) μm wide, containing eight ascospores arranged uniseriately or biseriately; base with croziers, apical apparatus urn-shaped, (4.6–)4.8–5.1(–5.4) × 3–3.2(–3.3) μm, euamyloid in Melzer’s reagent and Lugol’s solution. **Paraphyses** copious, filiform, thin-walled, equal to or longer than asci, 1.5–3 μm wide, somewhat septate, embedded in a gelatinous matrix, filled with large oily guttules best seen in fresh material. **Ascospores** (15.3–)16.5–17.6–19.1(–20.9) × (5.1–)5.5–6.2–6.8(–7.2) μm, Q = (2.4–)2.5–2.9–3.2(–3.5), N = 50, Ve = 358 μm^3^, ellipsoid–inequilateral, with broadly rounded or slightly pinched ends, occasionally aberrant with one acute end, cellular appendage not observed, dark olivaceous to medium brown, smooth, generally biguttulate, but also monoguttulate or with multiple guttules, germ slit straight, slightly shorter than spore length, located on the less convex side.

**Habitat and Distribution.** Gregarious, growing in groups on wood of *Laurus* spp. Known from laurel forests of the Canary Islands (La Gomera, Parque Nacional de Garajonay) and from continental laurel habitats in northern Portugal (Parque Nacional Peneda-Gerês).

**Typus.** Portugal, Braga, Terras de Bouro, Freitas, Parque Nacional Peneda-Gerês, N41°42′15.4″ W8°13′01.8″, 400 m a.s.l., growing in groups on *Laurus nobilis* wood, 16 February 2023, *leg.* A. Mateos & S. De la Peña, AMI-SPL1833.

**Additional material examined. Canary Islands.** Spain, Canary Islands, Santa Cruz de Tenerife, La Gomera, Parque Nacional de Garajonay, N28°06′37.3″, W17°14′54.5″, 1470 m a.s.l., growing in groups on *Laurus novocanariensis* wood, 5 December 2021, *leg.* A. Mateos, S. De la Peña & D. Chavez, AMI-SPL1119; ibid., AMI-SPL1121.

**Notes**. *Xylaria lauriphila* is recovered within the well-supported clade (SH-aLRT 100/BS 96) that includes *Xylaria polyphaga*, *X. conicoides*, *X. lauribaccicola*, *X. apiculata*, and *X. xylarioides*. This placement indicates its affinity with members of this group, although the phylogenetic analysis is not used here as the primary criterion for species delimitation.

*X. lauriphila* is characterized by its association with Lauraceae, occurring on woody substrates of this plant family, a feature that distinguishes it from most related taxa. In contrast, *X. polyphaga* exhibits a broad ecological amplitude and occurs on a wide variety of substrates, including both wood and fruits of different hosts (Table S1).

It differs from *X. lauribaccicola*, which is also associated with Lauraceae, by its occurrence on woody substrates rather than fruits, as well as by differences in stromatal morphology and micromorphological characters (see *X. lauribaccicola* in this paper). Additionally, *X. lauriphila* can be distinguished from *X. conicoides* by its substrate specificity and by differences in stromatal morphology, the latter species typically exhibiting a more distinctly conical fertile apex (Table S1).

From *X. xylarioides*, *X. lauriphila* differs in having more elongated and distinctly stipitate stromata, as well as in ecological preference, whereas *X. xylarioides* typically develops more compact stromata.

From a morphological standpoint, *Xylaria lauriphila* shares some characteristics with *X. arbuscula* var. *plenofissura*, but differs in having perithecial contours generally not exposed (only occasionally visible), an apical apparatus that is never tubular but uniformly urn-shaped, and larger ascospores, sometimes with slightly pinched ends, than those reported for that taxon ((13.7–)14.3–15.9(–17.7) × (4.8–)5.7–7.1(–7.4) μm, Me = 15.1 × 6.3 μm) [15]. In addition, the germ slit is somewhat shorter than the spore length and spore appendages are absent [9,11,17,51].

*Xylaria acuminata* is another morphologically similar species, but it differs in having stromata with perithecial contours never exposed, an apical apparatus variable from tubular to urn-shaped, and smaller ascospores (Me = 15.7 × 5.8 μm) compared to *X. lauriphila* (Me = 17.6 × 6.2 μm), as well as generally more broadly rounded spore ends (see *Xylaria acuminata* in this paper).

*Xylaria lauriphila* is distinguished by its association with Lauraceae wood, together with its stromatal morphology, ascospore characters, and other micromorphological features.

The combination of these ecological, macromorphological, and micromorphological characters allows its distinction from other currently recognized species within this group (Table S1).

***Xylaria peritheciata*** **A. Mateos & De la Peña-Lastra, sp. nov.**

**MycoBank No. 862987**

**Figure 19:**
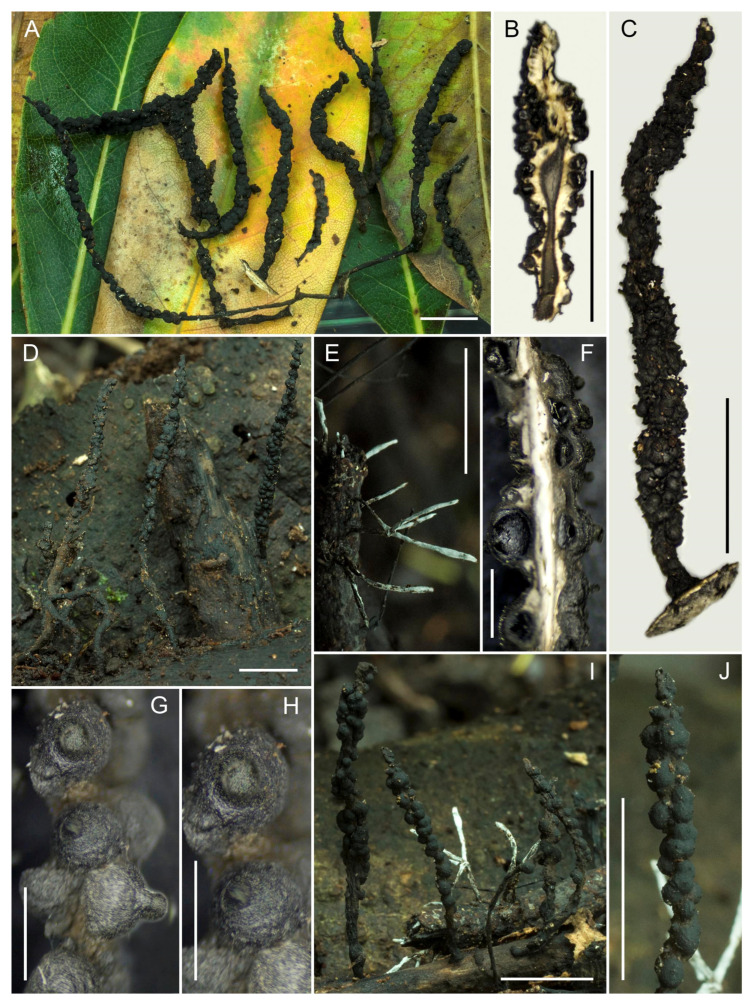
*Xylaria peritheciata*. Macromorphology. (**A**–**D**) AMI-SP1256 (Holotype). (**E**–**J**) AMI-SPL1258. (**A**) Morphological variability of the stromata. (**B**,**F**) Stroma in longitudinal section showing superficial perithecia and an interior white, soft spongy internal tissue. (**C**,**J**) Stroma showing a strongly nodulose surface. (**D**) Habit of mature stromata on the host natural substrate *Ocotea foetens* wood, in situ. (**E**) Habit of conidial stromata on host surface of *Laurus novocananiensis* wood, in situ. (**G**,**H**) Stromatal surface showing variously exposed perithecial contours, hemispherical to conical-papillated ostioles. (**I**) Mature teleomorphic stromata (black) with some white asexual (conidial) stromata, on *L. novocananiensis* wood. Scale bars: (**A**,**C**–**E**,**I**,**J**) = 10 mm, (**B**) = 5 mm, and (**F**–**H**) = 1 mm.

**Figure 20:**
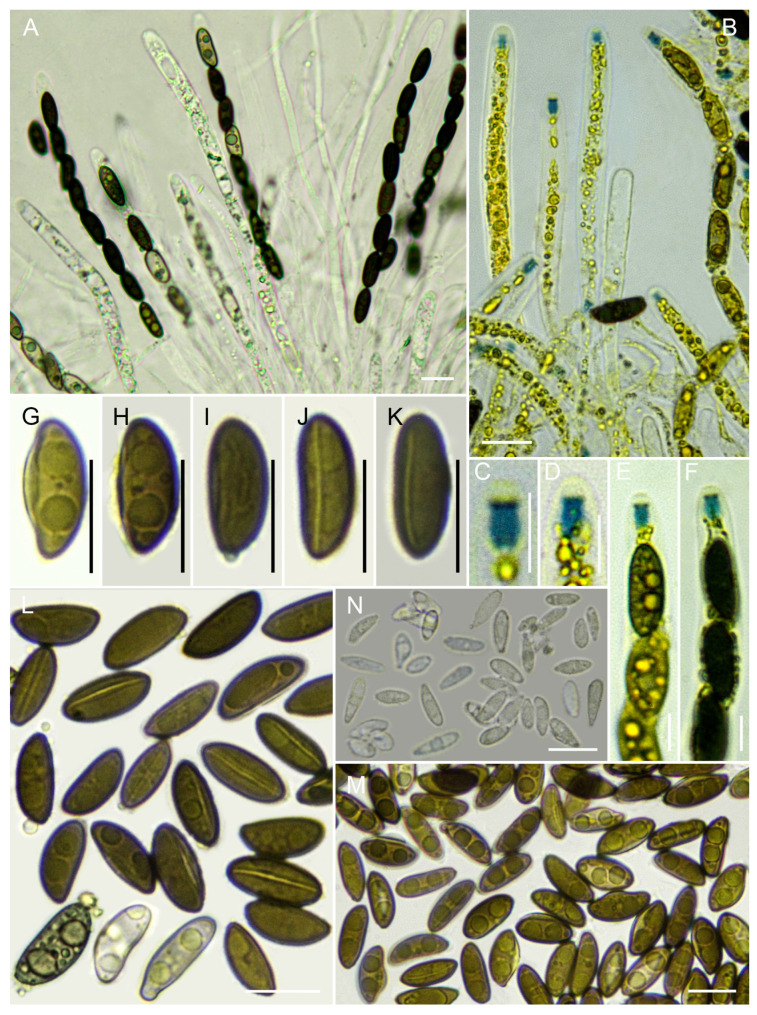
*Xylaria peritheciata*. Micromorphology. (**A**,**B**,**D**–**L**) AMI-SPL1256 (Holotype). (**C**,**M**,**N**) AMI-SPL1258. (**A**) Mature and immature asci with uniseriate ascospores, in water. (**B**) Mature and immature asci, in IKI-2 reagent. (**C**–**F**) Ascal apical apparati, in IKI-2 reagent. (**G**–**I**) Ascospore mucilaginous sheath and the basal cellular appendages observed. (**J**,**K**) Ascospores showing the germ slit, in water. (**L**,**M**) Mature and immature ascospores, with variously rounded ends, occasionally beaked, some showing a germ slit, in water. (**N**) Conidia clavate to pip-shaped, in water. Scale bars: (**A**,**B**,**G**–**N**) = 10 μm and (**C**–**F**) = 5 μm.

Figure 19 and Figure 20

**Etymology.** The specific epithet *peritheciata* derives from the Latin *perithecium*, referring to the flask-shaped ascomatal structures typical of many ascomycetes, combined with the suffix -*ata*, meaning “provided with” or “bearing.” The name refers to the conspicuous presence of perithecia in the stromata, whose contours are clearly visible and often prominently expressed on the stromatal surface.

**Teleomorph. Stromata** solitary to most often densely clustered, single or rarely branched in the fertile part, (25–)35–39(–82) mm in total height, erect, filiform and stipitate; fertile part (15–)31–35(–60) mm high × (1.5–)2.5–3 mm broad, terete, subcylindrical to fusiform, straight or somewhat curved, ending in a short sterile apex, 1–2.5 mm long, narrowly mucronate, fragile. Stromatal surface strongly nodulose, with perithecia partly immersed to 2/3 exposed or strongly exposed; outer crust black, rough, coriaceous, 14 μm thick, interior white, homogeneous, solid, spongy. **Stipe** sharply defined, short, 4–11 mm high × 0.5–1.0 mm diam., terete, glabrous, smooth or longitudinally ridged, slightly swollen at the base, surface black, leathery. **Perithecia** prominent, spherical to depressed-spherical, sometimes in contact, 450–750 μm high × 350–550 μm diam. **Ostioles** black, prominent, hemispherical to slightly conical-papillated, of up to 220 μm diam. at the base, conspicuous. **Anamorphic stromata** 10–12 mm high × 0.3–0.5 mm broad, fusiform, upright, with an acute apex, branched, powdery with conidia, white anamorphic layer gradually peeling off, the lower part is black and tomentose with a coupled base. **Asci** narrowly cylindrical, (95–)125–170(–190) μm in total length, spore-bearing part 75–110 μm long × 5–7 μm broad, containing eight uniseriately arranged ascospores; apical apparatus tubular, apically flattened with urn-shaped, 2–2.4 × 3–4 μm, euamyloid in Melzer’s reagent and Lugol’s solution. **Paraphyses** copious, filiform, thin-walled, equal to or longer than asci, 1.5–3.5 μm wide, somewhat septate, embedded in gelatinous matrix, filled with large oily guttules, best observed in fresh material. **Ascospores** (10.2–)11.1–12.1–13(–15.1) × (4.1–)4.3–4.9–5.3(–6.2) µm, Q = (2.1–)2.3–2.8(–3.1), N = 50, Qe = 2.5, Ve = 151 µm3, ellipsoid–inequilateral, with broadly rounded ends, rarely with a beaked end, sometimes bearing a minute hyaline cellular appendage up to 1.5 μm diam. at one end, or also with a mucilaginous sheath on the less convex side, dark olive-brown to medium brown, smooth, usually biguttulate though occasionally monoguttulate, with a conspicuous straight germ slit running the full length of the spore on the flattened ventral side. **Conidia** pip-shaped, fusiform, 8–9.5 × 2.5–3.2 μm, hyaline, smooth.

**Habitat and Distribution.** Gregarious, occurring in laurel forest habitats, growing in groups on wood of *Ocotea foetens*. Known from the Azores.

**Typus.** Portugal, Azores, Terceira, Praia da Vitória, Fontinhas, Largo São João, 38°45′17.9″ N 27°07′30.2″ W, 0 m a.s.l., growing in groups on *Ocotea foetens* wood, 14 January 2022, *leg.* A. Mateos & S. De la Peña, AMI-SPL1256.

**Additional material examined.** Portugal, Azores, Terceira, Praia da Vitória, Fontinhas, Largo São João, 38°45′17.9″ N 27°07′30.2″ W, 0 m a.s.l., growing in groups on *Ocotea foetens* wood, 14 January 2022, *leg.* A. Mateos & S. De la Peña, AMI-SPL1280; *ibid.*, AMI-SPL1258, AMI-SPL1276, AMI-SPL1277, AMI-SPL1278, AMI-SPL1279, AMI-SPL1291.

**Notes**. *Xylaria peritheciata* forms a distinct and well-supported monophyletic lineage (SH-aLRT 90/BS 99) in the present phylogenetic analyses. This lineage is recovered within a broader, well-supported clade (SH-aLRT 100/BS 100) that also comprises *Xylaria violaceorosea*. Its position in this clade clearly separates it from the nearby *X. cinerea*–*X. oligotoma* group.

*X. peritheciata* is characterized by simple, filiform stromata with strongly developed, conspicuous perithecial mounds and ostioles, giving the fertile surface a markedly uneven and nodulose appearance. This feature readily distinguishes it from *X. filiformis*, which typically exhibits more slender, smoother stromata with less evident perithecial contours.

It also differs from *X. violaceorosea*, which is readily recognized by its violaceous to pinkish stromatal pigmentation, by lacking any violet or vinaceous outer layer and by having a much more strongly perithecioid, nodulose stromatal surface. In addition, *X. peritheciata* differs from *X. filiformis* and related taxa in stromatal robustness and external morphology, presenting thicker and more irregular fertile parts, whereas *X. filiformis* is generally more filiform and regularly shaped. Differences in ascospore dimensions and morphology further support their separation.

Other species with similarly nodulose stromata include *Xylaria delitschii*, which, however, shows less prominent perithecia, differs in having smaller ostioles, a cuboid (not urn-shaped) apical apparatus, and smaller ascospores ((9.5–)10.4–11.9(–12.8) × (4.3–)4.6–5.2(–5.3) μm) with somewhat acute ends [8]; moreover, its anamorph is whitish with orange areas. *Xylaria oxyacanthae*, like *X. delitschii*, has perithecia that are not as prominent as those of *X. peritheciata*, and differs in color (pale golden brown), possesses a cuboid apical apparatus, and has smaller ascospores ((9.0–)10.1–11.4(–12.0) × (4.3–)4.7–5.2(–5.4) μm) with slightly acute ends and without visible sheath or appendages, and its anamorphic stromata are filiform with acute apices [8].

*Xylaria vasconica* presents a cerebriform aspect with wrinkles encompassing several perithecia together rather than individually, as in *X. peritheciata*. In addition, its perithecia are more immersed and less prominent, its germ slit is somewhat shorter, and its anamorphic conidia are distinctly fusiform. *Xylaria karsticola* is morphologically closer to the latter species [8,65].

*Xylaria* cf. *gracillima* differs by having smaller stromata, smaller asci, a short cylindrical apical apparatus, and smaller ascospores ((9–)10.3–12(–13.1) × (3.1–)3.6–4.5(–4.9) µm), ellipsoid–inequilateral, with a much shorter germ slit and lacking appendages or mucilaginous sheath [15]. *Xylaria schwackei* differs in having stromata with grayish-olive to light brown scales at the base of the perithecial mounds and blunt, scarcely prominent ostioles [15]. *Xylaria warburgii*, described from Taiwan, is distinct in having small, papillate, slightly prominent ostioles and smaller ascospores [66].

The combination of its distinct stromatal morphology, especially the strongly expressed perithecial mounds, together with its independent phylogenetic position, supports the recognition of *Xylaria peritheciata* as a distinct species.

***Xylaria polymorpha* (Pers.) Grev., Fl. Edin.: 355 (1824)**

**Figure 21:**
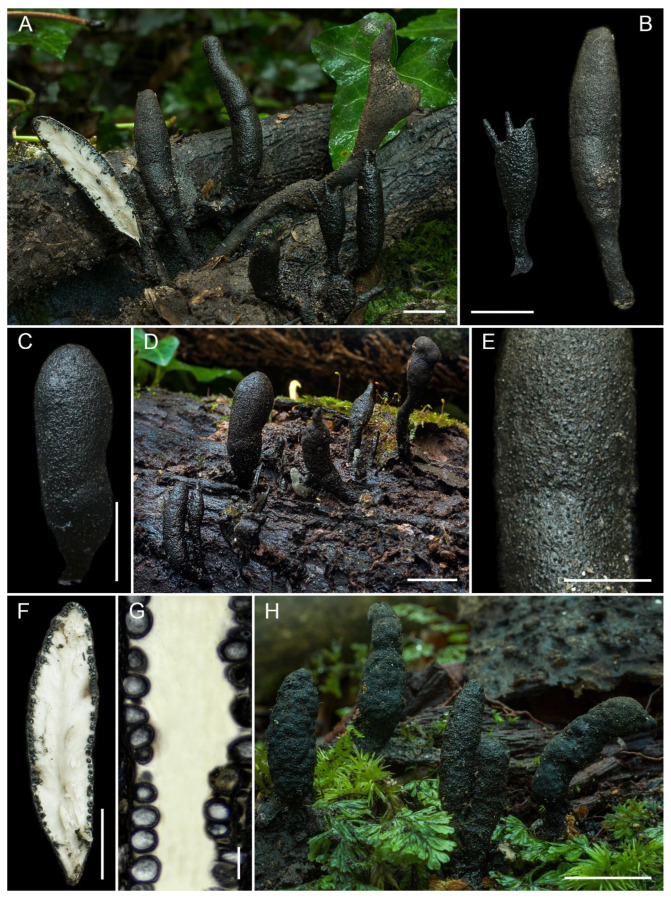
*Xylaria polymorpha*. Macromorphology. (**A**,**B**,**E**,**F**) AMI-SPL1533. (**C**,**D**,**G**) AMI-SPL1586. (**H**) AMI-SPL764. (**A**–**D**,**H**) Morphological variability of the mature stromata on the host natural substrate *Laurus nobilis* wood, in situ. (**C**,**E**) Stromatal surface and ostioles. (**F**,**G**) Perithecia vertical section fully immersed in a white fibrous internal tissue. Scale bars: (**A**–**D**,**F**,**H**) = 10 mm, (**E**) = 5 mm, and (**G**) = 1 mm.

**Figure 22:**
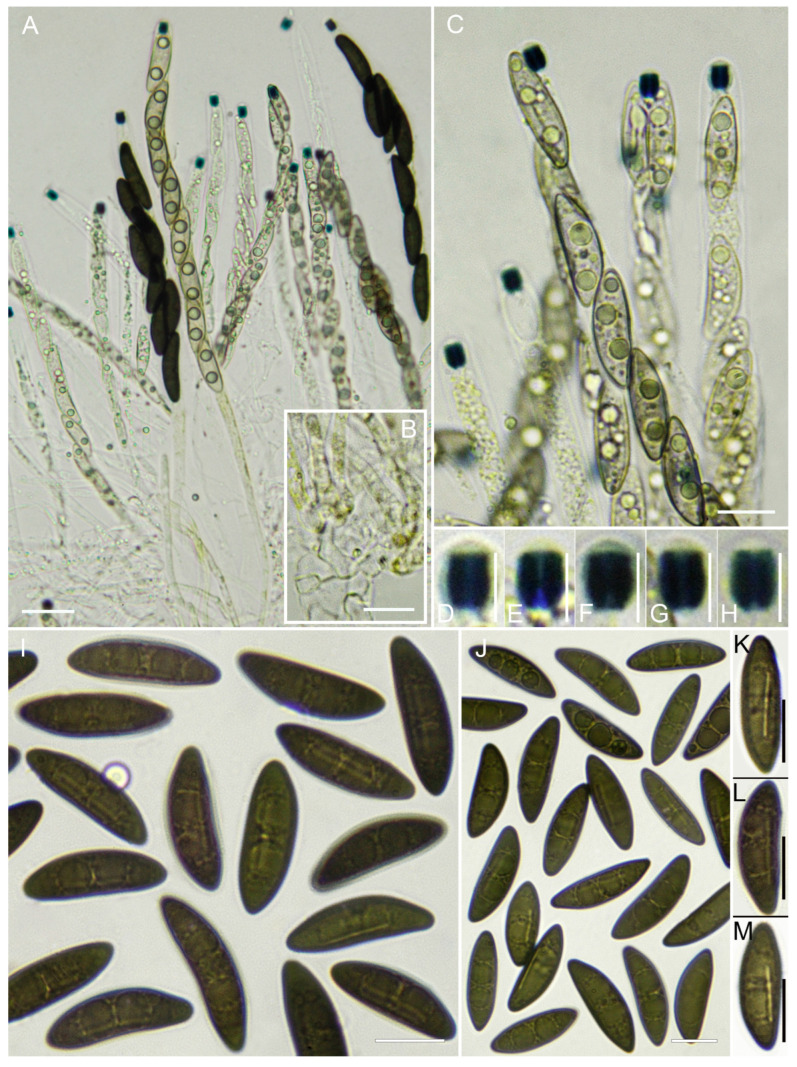
*Xylaria polymorpha*. Micromorphology. (**A**–**I**,**K**–**M**) AMI-SPL1586. (**J**) AMI-SPL764. (**A**,**C**) Mature and immature asci in IKI-2 reagent. (**B**) Asci with details of the base, in water. (**D**–**H**) Ascal apical apparati in IKI-2 reagent. (**I**,**J**) Mature and immature ascospores in water. (**K**–**M**) Ascospores in ventral view showing the germ slit, in water. Scale bars: (**A**–**C**,**I**–**M**) = 10 μm and (**D**–**H**) = 5 μm.

Figure 21 and Figure 22

**Teleomorph**. **Stromata** highly variable, solitary but most often clustered, simple or very rarely branched, (23–)26–60(–95) mm total height, erect, stipitate; fertile portion terete, 18–24(–45) mm high × (4–)5–10(–14) mm broad, clavate, subcylindrical–clavate, ellipsoid, fusiform or spatulate, straight or recurved, occasionally terminating in one or several fertile apices 1.2–4 mm long, rounded or conical–mucronate, fragile. Surface rough to very rough, cracked into small polygonal scales, dark brown, gray–green to black; cortex up to 40–50 μm thick, hard-textured, leathery; interior white, becoming yellowish in the medulla at maturity, homogeneous, soft-spongy. **Stipe** short or long, sometimes poorly differentiated and gradually merging with the fertile portion, 3–45(–50) mm high × 1.5–6 mm diam., terete, rarely bifurcate, occasionally long-radicating, surface black, leathery. **Perithecia** immersed, little or not exposed, spherical to subglobose, 600–1000 μm high × 550–950 μm diam. **Ostioles** abundant, black, hemispherical to raised-discoid (to 230 μm), papillate at the center. **Asci** narrowly cylindrical, (160–)205.5–212.2(–265) μm total length, with a spore-bearing portion 109–150 μm long × (7.2–)7.7–10.3(–12.3) μm wide, containing eight ascospores arranged uniseriately, sometimes slightly overlapping, base aporhynchous with short multiseptate elements, apical apparatus rectangular to slightly urn-shaped, (3.1–)3.3–3.4(–3.5) × (3.9–)4–4.3(–4.4) μm, euamyloid in Melzer’s reagent and Lugol’s solution. **Paraphyses** copious, filiform, thin-walled, equal to or longer than the asci, 1–2.5(–4) μm wide, somewhat septate, embedded in a gelatinous matrix, filled with large oily guttules best seen in fresh material. **Ascospores** (18.6–)20.6–22.1–23.9(–25.3) × (5.6–)6.1–6.6–7.3(–7.5) μm, Q = (2.6–)3–3.3–3.7(–4.2), N = 50, Ve = 512 μm^3^, ellipsoid–inequilateral to navicular, sometimes slightly curved, with rounded to slightly pinched ends, cellular appendage not observed, dark olive in vivo, becoming brown, smooth, generally biguttulate, sometimes with 3–4 guttules, germ slit straight or slightly oblique, 1/2–2/3 of the spore length, located on the less convex side.

**Notes**. *Xylaria polymorpha* is apparently easy to recognize because of its large size and peculiar shape; however, it exhibits considerable morphological variability, hence its name, and may, therefore, be confused with other species. According to Rogers and Callan [67], it is probably the most frequently cited *Xylaria* species worldwide and represents a taxonomically complex entity, encompassing forms and sizes similar to numerous other taxa from which it may be difficult to distinguish.

Closely related taxa, according to Rogers and Callan [67], include *Xylaria schweinitzii*, which differs from *X. polymorpha* in having few or no ascospores with a straight germ slit, the slit being usually oblique, curved or spiral [67,68]; *Xylaria grandis*, with somewhat smaller ascospores (20–23 × 7 μm) and a germ slit extending for 2/3 or more of the ascospore length [67], and *Xylaria scruposa*, characterized by smaller ascospores (16–22 μm long) and a germ slit that is straight, oblique, or slightly sigmoid [14,67,69].

Two additional species occurring in the same European geographical area, *Xylaria corniformis* and *Xylaria longipes* [8], present a superficially similar stromatal surface but are readily distinguished from *X. polymorpha* by their much smaller ascospores in both cases, and in the latter species, also by the sigmoid morphology of the germ slit [8,70].

*Xylaria digitata*, cited in some classical works as *X. polymorpha*, clearly differs in possessing a sterile stromatal apex [71,72]. *Xylaria cubensis*, belonging to the *X. polymorpha* group, is readily distinguished from other members of this group by its thick leathery cortex [8]; moreover, it differs markedly from the type species in its brown stromatal color and in having ascospores that are very different in shape and considerably smaller [56].

**Specimens examined. Cortegada Islands.** Spain, Galicia, Pontevedra, Parque Nacional de las Islas Atlánticas de Galicia, Cortegada Island, 42°37′10.29″ N, 8°47′16.85″ W, 4 m a.s.l., growing in groups on *Laurus nobilis* wood, 10 November 2022, *leg.* A. Mateos & S. De la Peña, AMI-SPL1533; *ibid.*, AMI-SPL1586. **Madeira Islands.** Portugal, Madeira, São Vicente, Chão dos Louros, 32°45′37.1″ N, 17°00′58.5″ W, 820 m a.s.l., growing in groups on *Laurus novocanariensis* wood, 19 November 2021, *leg.* A. Mateos & S. De la Peña, AMI-SPL764.

***Xylaria polyphaga*** **De la Peña-Lastra & A. Mateos, sp. nov.**

**MycoBank No. 862986**

**Figure 23:**
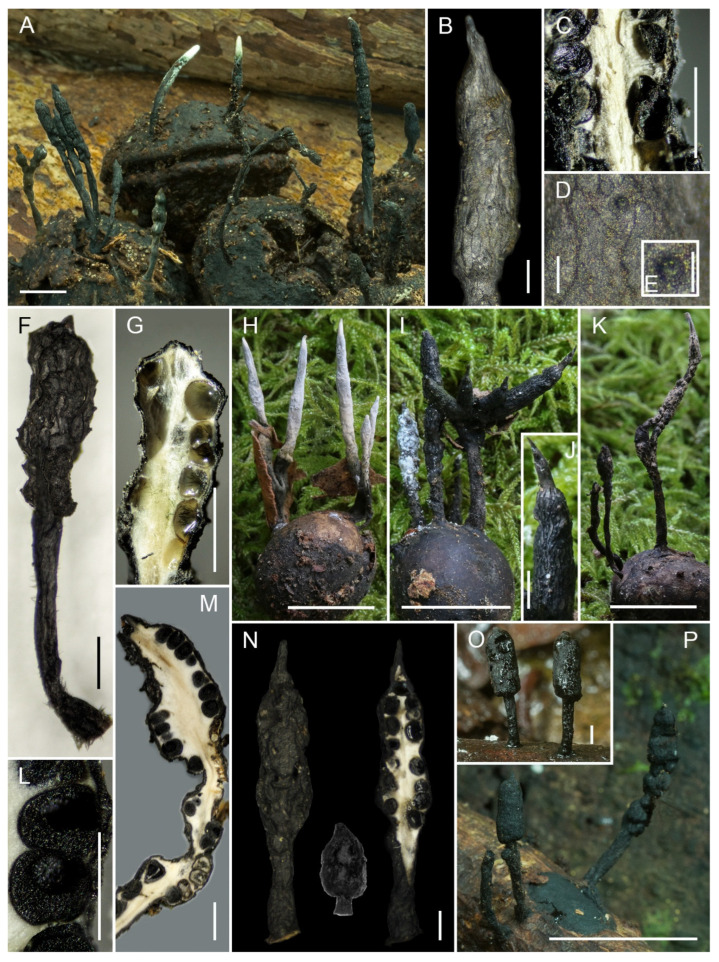
*Xylaria polyphaga*. Macromorphology. (**A**–**E**) AMI-SP1254 (Holotype). (**F**,**G**) AMI-SPL1657. (**H**–**K**) AMI-SPL1167. (**L**,**M**,**P**) AMI-SPL1257. (**N**,**O**) AMI-SPL925. (**A**) Mature teleomorph stromata, some with asexual stromata a conidial white apex on host surface of *Eucalyptus globulus* fruits, in situ. (**B**,**F**,**N**) Mature stroma, subcylindrical, showing a sterile apiculate apex and a slightly nodulose grayish to blackish outer layer, on a hairy stipe. (**D**,**E**) Stromatal surface and ostioles. (**C**,**G**,**L**–**N**) Stroma in vertical section showing perithecia fully immersed in a white fibrous internal tissue, with a ochreous inner core. (**H**–**K**) Habit of conidial stromata (**H**), habit of mature teleomorphic stromata (**I**,**J**), and habit of semi-mature (**K**), on host surface of *Laurus nobilis* berries, in situ. (**O**,**P**) Morphological variability of the stromata on the natural substrate of *Ocotea foetens* wood. Scale bars: (**A**,**H**,**I**,**K**,**P**) = 10 mm, (**B**,**C**,**F**,**G**,**J**,**M**–**O**) = 1 mm, (**D**,**L**) = 0.5 mm, and (**E**) = 0.2 mm.

**Figure 24:**
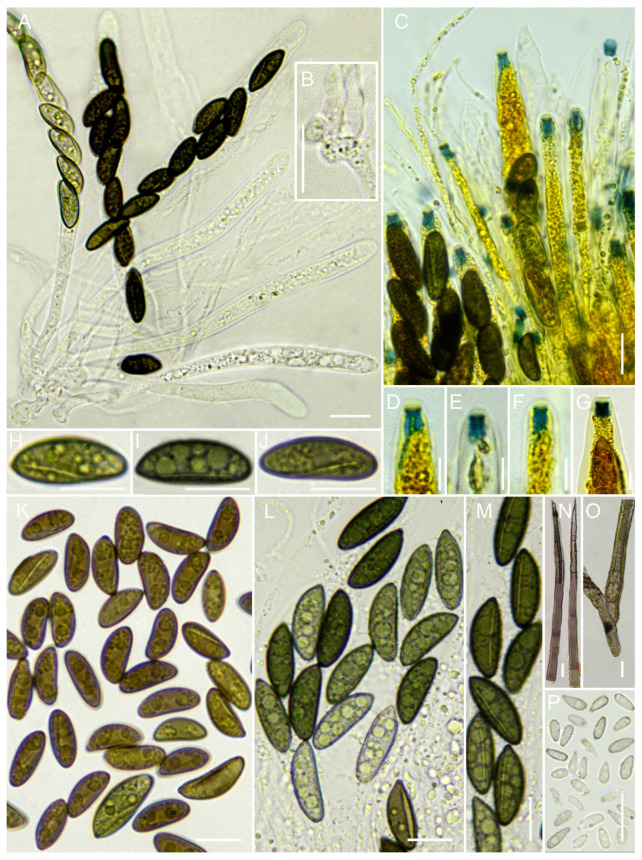
*Xylaria polyphaga*. Micromorphology. (**A**–**F**,**H**–**K**) AMI-SPL1254 (Holotype). (**G**,**L**,**M**,**P**) AMI-SPL925. (**N**,**O**) AMI-SPL2003. (**A**) Mature and immature asci with uniseriate ascospores, showing details of the base, in water. (**B**) Ascal bases with croziers. (**C**) Mature and immature asci, with paraphyses containing refractive guttules, in IKI-2 reagent. (**D**–**G**) Ascal apical apparati, in IKI-2 reagent. (**H**–**J**) Ascospores showing the germ slit, in water. (**K**,**L**) Mature and immature ascospores in water. (**M**) Ascospores in ventral view showing the germ slit, in water. (**N**) Apex of setae. (**O**) Base of setae. (**P**) Conidia, in water. Scale bars: (**A**–**C**,**H**–**O**) = 10 μm and (**D**–**G**,**P**) = 5 μm.

Figure 23 and Figure 24

**Etymology.** The specific epithet *polyphaga* derives from the Greek *poly-*(πολύς), meaning “many” or “much” and -*phagos* (φαγός), meaning “feeding on” or “consuming.” The name refers to the broad ecological amplitude of the species, reflecting its ability to colonize a wide range of substrates, from fruits of different plant species to the wood of various trees.

**Teleomorph. Stromata** solitary to most often densely clustered, simple or rarely branched above the stipe, (8–)15–20(–27) mm total height, erect, stipitate, fertile parts 8–16 mm high × 1.2–2 mm broad, terete, subcylindrical to narrowly fusiform, straight with narrow, fragile, rounded, or mucronate, sterile extension 1–2.5 mm long apically ending, with longitudinally oriented scales, strips, and plates delimiting narrow cracks. Surface grayish to blackish, hard-textured, leathery, slightly nodulose with perithecial contours not too faintly exposed, more evident in the lower part, 30–70(–90) μm thick, inside white, homogeneous, and soft spongy. **Stipe** sharply defined, 4–11 mm high × 0.5–1.0 mm diam., terete, slightly swollen at base, subsurface black, leathery, densely covered in short, light blackish-brown hairs, abundant when young and disappearing at maturity, 50–400 × 5–8 μm, septate. **Perithecia** spherical to depressed spherical 700–800 μm high × 500–700 μm diam. at the base. **Ostioles** black, blunt papilla, up to 120 μm diam., often inconspicuous. **Anamorphic stromata** 19–24 mm high × 0.8–1.2 mm broad, subcylindrical to fusiform, upright, apex either tapered, unbranched, powdery with conidia, white anamorphic layer peeling off gradually, revealing the underlying gray-greenish brown layer, lower part black and tomentose, with flared and coupled base. **Asci** narrowly cylindrical, (80–)90–140(–153) μm total length, spore-bearing parts 55–84 μm long × 5.5–7 μm broad, containing eight ascospores arranged uniseriately, sometimes slightly overlapping, base pleurorhynchous, with clamp connections; apical apparatus tubular, apically flattened with urn-shaped with a slightly widened apex, 2.2–3.5 × 3.5–4 μm, euamyloid in Melzer’s reagent and Lugol’s solution. **Paraphyses** abundant, filiform, longer than the asci, 1–3 μm wide, somewhat septate, filled with large oily guttules, best observed in fresh material. **Ascospores** (10.9–)11.6–13.8–17.3(–18.5) × (4.7–)5–5.8–6.8(–7.2) µm, Q = (1.7–)2.2–2.8(–3), N = 50, Qe = 2.45, Ve = 255 µm3, ellipsoid–inequilateral, with broadly rounded ends, rarely with one end acute, cellular appendage not observed, dark olive brown to medium brown, smooth, bigutulate, with a conspicuous straight, sometimes sigmoid, germ slit 3/4 spore length on the flattened ventral side. Setae on the stipe thick-walled, with an acuminate apex and bifurcate base, 50–100 μm long × 5–10 μm wide. **Conidia** fusiform–clavate, 4–6 × 2.3–2.8 μm, hyaline, smooth.

**Habitat and Distribution.** Gregarious, occurring in more or less numerous groups in laurel forest habitats, growing on dead wood and fruits of *Laurus* spp., as well as on fruits of *Eucalyptus*. Known from different and geographically distant laurel forest localities, both continental and insular.

**Typus.** Portugal, Azores, Terceira, Praia da Vitória, Fontinhas, Largo São João, 38°45′17.9″ N 27°07′30.2″ W, 0 m a.s.l., growing gregariously on the fruits of *Eucalyptus globulus*, 14 January 2022, *leg.* A. Mateos & S. De la Peña, AMI-SPL1254.

**Additional material examined. Cortegada Islands.** Spain, Galicia, Pontevedra, Parque Nacional de las Islas Atlánticas de Galicia, Cortegada Island, 42°37′10.29″ N, 8°47′16.85″ W, 4 m a.s.l., growing gregariously on the fruits of *Laurus nobilis*, 28 December 2021, *leg.* A. Mateos & S. De la Peña, AMI-SPL1167. Spain, Galicia, Pontevedra, Parque Nacional de las Islas Atlánticas de Galicia, Cortegada Island, 42°37′10.29″ N, 8°47′16.85″ W, 4 m a.s.l., growing in groups on *L. nobilis* wood, 31 December 2016, *leg.* A. Mateos & S. De la Peña, AMI-SPL2003. Spain, Galicia, Pontevedra, Parque Nacional de las Islas Atlánticas de Galicia, Cortegada Island, 42°36′57.5″ N, 8°47′05.3″ W, 2 m a.s.l., growing gregariously on the fruits of *L. nobilis*, 9 November 2015, *leg.* A. Mateos & S. De la Peña, AMI-SPL2021. Spain, Galicia, Pontevedra, Parque Nacional de las Islas Atlánticas de Galicia, Cortegada Island, 42°36′57.5″ N, 8°47′05.3″ W, 2 m a.s.l., growing in groups on *L. nobilis* wood, 15 November 2017, *leg.* A. Mateos & S. De la Peña, AMI-SPL2024. **Azores Islands.** Portugal, Azores, Terceira, Praia da Vitória, Fontinhas, Largo São João, 38°45′17.9″ N 27°07′30.2″ W, 0 m a.s.l., growing in groups on *Laurus azorica* wood, 14 January 2022, *leg.* A. Mateos & S. De la Peña, AMI-SPL1257. **Parque Natural Los Alcornocales.** Spain, Cádiz, Los Barrios, Las Corzas, Parque Natural Los Alcornocales, 36°08′11.17″ N 5°31′11.46″ W, 353 m a.s.l., growing in groups on *Laurus nobilis* wood, 17 December 2022, *leg. A. Mateos*, *S. De la Peña & M. Plaza*, AMI-SPL1657. **Canary Islands.** Spain, Canary Islands, Santa Cruz de Tenerife, Monte de las Mercedes, Parque Rural de Anaga, 28°31′49.26″ N 16°17′09.67″ W, 922 m a.s.l., growing in groups on *Laurus novocanariensis* wood, 2 December 2021, *leg.* A. Mateos & S. De la Peña, AMI-SPL925.

**Notes**. *Xylaria polyphaga* is recovered within the well-supported clade (SH-aLRT 100/BS 96) that includes *X. conicoides*, *X. lauribaccicola*, *X. lauriphila*, *X. apiculata*, and *X. xylarioides*. This placement indicates its affinity with members of this group, although the phylogenetic analysis is not used here as the primary criterion for species delimitation. It is clearly distinct from members of the *X. arbuscula* complex, with which it shares certain superficial similarities in stromatal habit.

From a morphological standpoint, *Xylaria polyphaga* shares several features with members of the *X. arbuscula* complex, both in its growth habit and macroscopic appearance, having been reported on various hosts, such as wood, pods, or fructifications (Fournier et al., 2020); however, it differs in having smaller ascospores ((12.0–)12.9–14.1(–14.9) × (4.9–)5.2–5.7(–5.9) μm) and a larger apical apparatus [8,15]. In comparison with *Xylaria arbuscula* var. *plenofissura*, it shows greater divergence, as the latter possesses larger asci, and notably, larger ascospores with a longer germ slit, almost reaching the full length of the spore [15].

*X. polyphaga* is characterized by its subcylindrical to narrowly fusiform, stipitate stromata with a short mucronate sterile apex, a stromatal surface bearing longitudinal scales and plates, and a white internal tissue with an ochreous inner core. It produces relatively small ascospores and a comparatively large apical apparatus, and is notable for its broad ecological amplitude, occurring on a wide range of substrates, including wood and fruits of different host plants.

Within its clade, *X. polyphaga* can be distinguished from *X. conicoides* by having larger ascospores, less distinctly conical stromata, and a markedly broader ecological amplitude. From *X. lauriphila* it differs in its polyphagous ecology, larger ascospores, and stromatal surface with longitudinal scales and plates. It is further separated from *X. xylarioides* by its distinctly stipitate and elongated stromata, as opposed to the more compact, subglobose to conical forms observed in that species, as well as by the presence of an ochreous inner core (Table S1).

Although sharing some superficial resemblance with members of the *X. arbuscula* complex, *X. polyphaga* is clearly distinct both phylogenetically and morphologically, differing in ascospore size, apical apparatus dimensions, and ecological breadth.

The combination of molecular phylogenetic evidence and morphological differences supports the recognition of *Xylaria polyphaga* as a distinct species (Table S1).

***Xylaria violaceorosea*** **J. Fourn., A. Román, Balda & E. Rubio, Ascomycete.org 6(2): 35 (2014)**

**Figure 25:**
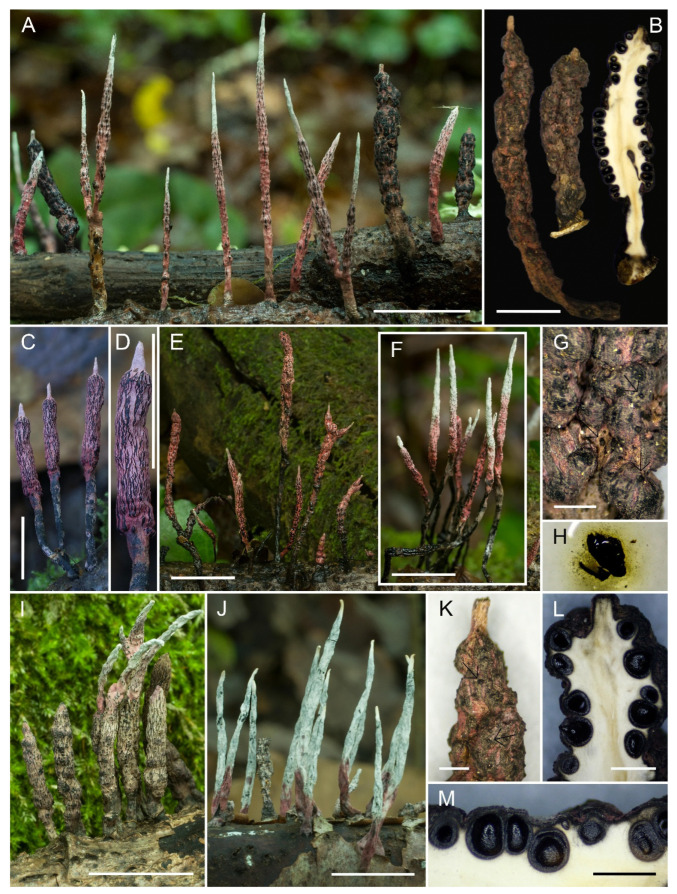
*Xylaria violaceorosea*. Macromorphology. (**A**,**B**,**G**,**H**,**K**–**M**) AMI-SP1479. (**C**,**D**) AMI-SPL569. (**E**,**F**) AMI-SPL1565. (**I**,**J**) AMI-SPL1671. (**A**) Mature and immature teleomorph stromata, on host surface of *Quercus canariensis*, in situ. (**B**,**E**) Morphological variability of the mature stromata. (**I**) Mature teleomorph stromata on which anamorphs grow in a vegetative growth sequence, teratological form. (**C**,**D**,**F**) Immature stromata. (**J**) Habit of anamorphic stromata, on host surface of *Q. canariensis*, in situ. (**G**,**K**) Stromatal surface with ostioles. (**B**,**L**,**M**) Stroma in vertical section showing perithecia fully immersed in a white fibrous internal tissue. (**H**) 10% KOH, a fragment of the outer layer produces olive-yellow pigments. Scale bars: (**A**,**C**–**F**,**I**,**J**) = 10 mm, (**B**) = 5 mm, and (**G**,**K**–**M**) = 1 mm.

**Figure 26:**
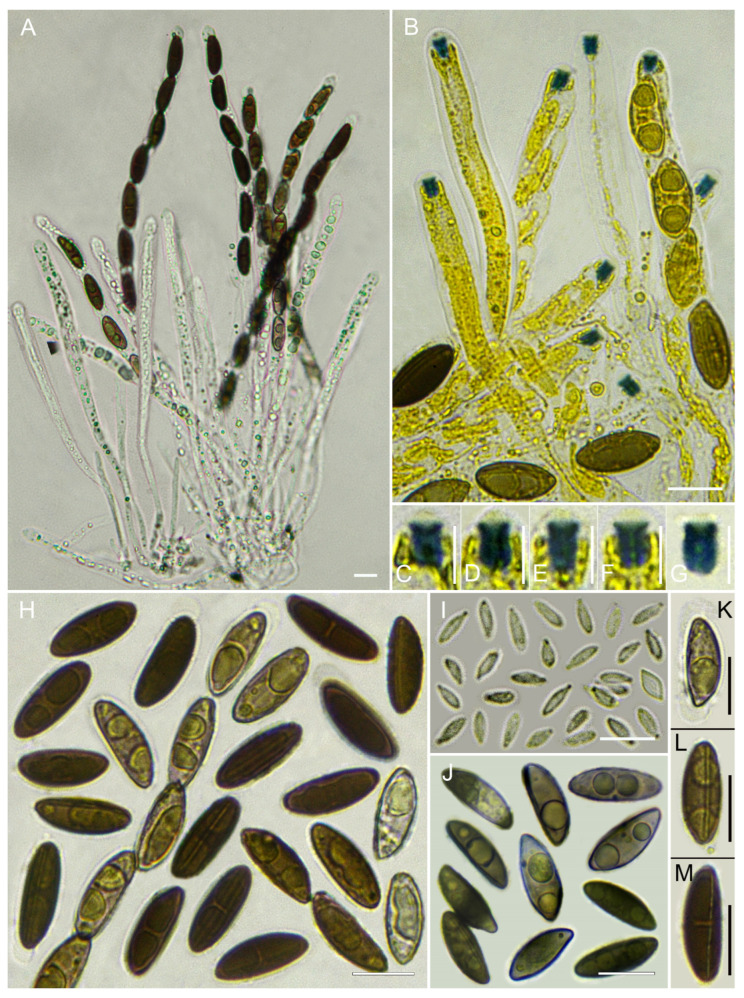
*Xylaria violaceorosea*. Micromorphology. (**A**–**H**,**J**–**M**) AMI-SP1479. (**I**) AMI-SPL1671. (**A**) Mature and immature asci with uniseriate ascospores, in water. (**B**) Mature and immature asci, in IKI-2 reagent. (**C**–**G**) Ascal apical apparati, in IKI-2 reagent. (**H**,**J**) Mature and immature ascospores in water. (**K**) Ascospore showing a mucilaginous sheath stained visible in Indian ink. (**L**,**M**) Ascospores showing the germ slit, in water. (**I**) Conidia clavate to pip-shaped, in water. Scale bars: (**A**,**B**,**H**–**M**) = 10 μm and (**C**–**G**) = 5 μm.

Figure 25 and Figure 26

**Teleomorph**. **Stromata** solitary, often grouped and sometimes densely gregarious or even fasciculate, simple or more rarely bifurcate either in the fertile portion or at the level of the stipe, immature specimens are more elongated and narrowly stylized, reaching 14–40 mm total height, mature stromata 11–27 mm total height, erect, stipitate; fertile portion 14–21 mm high × 1–3.2 mm broad, terete, cylindrical to subcylindrical, straight, terminating in a short mucronate apex 1–2 mm long, sometimes rounded, fragile. Surface nodulose, with deep wrinkles and perithecial contours slightly to strongly exposed; an outer layer colored purple to vinaceous purple and sometimes pink, forms elongated vertical strips that fade and disappear with age, between them appear grayish to black fissures; aging specimens become dark grayish to blackish, often retaining purplish-pink tones in some areas; cortex up to 50–60(–70) μm thick, hard-textured, leathery; in 10% KOH, after more than 1 min, fragments of the outer layer turn olivaceous yellow; interior white, becoming yellowish in the medulla with age, homogeneous, caseous to somewhat fibrillose. **Stipe** sometimes subsessile or very short but generally well defined, cylindrical, 4–6(–11) mm high × 1–2 mm diam., terete, widened at the base where attached to the substrate (–2 mm), subsurface concolorous with the rest, becoming blackish with age, leathery. **Perithecia** generally immersed, occasionally slightly exposed, spherical to subglobose, 580–750 μm high × 540–630 μm diam. **Ostioles** black, conical–papillate, up to 80 μm diam., located among the gray strips. **Asci** narrowly cylindrical, (130–)180–196(–208) μm total length, with a spore-bearing portion (99–)109.5–120 μm long × (6.1–)6.2–7.5(–8) μm wide, containing eight ascospores arranged uniseriately, base aporhynchous, apical apparatus tubular with a rim at the upper end, (3.3–)3.5–3.85(–3.9) × (2.2–)2.4(–2.6) μm, euamyloid in Melzer’s reagent and Lugol’s solution. **Paraphyses** abundant, filiform, thin-walled, equal in length to the asci, 1–2 μm wide, somewhat septate, containing oily guttules, embedded in a gelatinous matrix. **Ascospores** (14.7–)15.3–16–16.8(–17.7) × (5–)5.4–5.7–6(–6.4) μm, Q = (2.5–)2.6–2.8–3(–3.2), N = 50, Ve = 271 μm^3^, fusiform, somewhat inequilateral, with broadly rounded ends, occasionally with one acute end and the other rounded, rarely with a sharply pointed or mucronate end, with mucilaginous appendages at both ends connected along the less convex side and bearing a fugacious hyaline cellular appendage, brown to olivaceous-brown, smooth, mono- to bi-guttulate, germ slit straight, parallel to the long axis or slightly diagonal, extending the full length of the spore on the less convex side. **Anamorphic stromata** (AMI-SPL1671) solitary or generally gregarious, sometimes intermixed with mature teleomorphs or even remaining on their apex, erect, filiform, upright, thread-like, (15–)25–40 × 1–1.5(–2) mm. Underlying layer black, covered by a whitish pulverulent pruina, ending in an acute apex, not bifurcate, base attached to the substrate, violaceous–vinaceous in color. Conidiogenous layer forming a dense palisade, with terminal cylindrical conidiogenous cells, 15–30 × 2.5–3.5 μm, hyaline to slightly grayish-brown. **Conidia** produced holoblastically in sympodial succession, hyaline, smooth, oval to obclavate, fusiform–clavate, clavate to pip-shaped, (3.7–)4.2–6(–7) × (1.5–)1.7–2.1(–2.2) μm, with truncate bases indicating former points of attachment to conidiogenous cells. Conidia germinating in their natural habitat.

**Notes**. Despite the striking color of this species and its locally abundant growth, it was surprisingly not described until relatively recently [8]. As indicated in that publication (*in opus cit.*) the violet-pink coloration is not entirely unknown in species of the genus *Xylaria* spp., but it had not previously been reported from temperate regions. In contrast, several species with similar pigmentation have been described from tropical areas, for example, *Xylaria ianthino-velutina*, described from Taiwan, also shows a violaceous coloration but lacks the pink tones observed in *X. violaceorosea*—it occurs on woody pods and produces smaller ascospores measuring 9–12 × 3.5–5 μm [17,73]. *Xylaria violaceo-pannosa*, described from Paraguay, differs in having flattened-clavate stromata of dark violet color with a verrucose to rough surface, and much smaller ascospores (10–11 × 5–5.5 μm) [74]. *Xylaria moliwensis*, described from Cameroon, has clavate pink stromata and considerably larger ascospores (mean 36.2 × 8.8 μm) than those of *X. violaceorosea*, and lacks secondary appendages [21]. *Xylaria rosea*, collected in the Congo (Zaire), is known from conidial stromata that are cylindrical–clavate with broadly rounded apices [21,75].

In addition to the localities reported in the original description from several provinces of northern Spain, *X. violaceorosea* has subsequently been recorded in nearby regions [29,76,77,78,79], as well as in Portugal and Mexico [80], generally in areas with subtropical climatic influence. The present study extends its distribution to southern Spain (see material examined in this work).

**Specimens examined. Cortegada Islands.** Spain, Galicia, Pontevedra, Parque Nacional de las Islas Atlánticas de Galicia, Cortegada Island, 42°36′57.5″ N, 8°47′05.3″ W, 2 m a.s.l., growing in groups on *Quercus robur* wood, 9 November 2022, *leg.* A. Mateos & S. De la Peña, AMI-SPL1479. Spain, Galicia, Pontevedra, Parque Nacional de las Islas Atlánticas de Galicia, Cortegada Island, 42°37′10.29″ N, 8°47′16.85″ W, 4 m a.s.l., growing in groups on *Q. robur* wood, 10 November 2022, *leg.* A. Mateos & S. De la Peña, AMI-SPL1565. Spain, Galicia, Pontevedra, Parque Nacional de las Islas Atlánticas de Galicia, Cortegada Island, 42°36′56.1″ N, 8°47′13.5″ W, 4 m a.s.l., growing in groups on *Q. robur* wood, 15 November 2017, *leg.* A. Mateos & S. De la Peña, AMI-SPL2025; *ibid.*, AMI-SPL2027. Spain, Galicia, Pontevedra, Parque Nacional de las Islas Atlánticas de Galicia, Cortegada Island, 42°37′10.29″ N, 8°47′16.85″ W, 4 m a.s.l., growing in groups on *Q. robur* wood, 27 November 2020, *leg.* A. Mateos & S. De la Peña, AMI-SPL569. Spain, Galicia, Pontevedra, Parque Nacional de las Islas Atlánticas de Galicia, Cortegada Island, 42°37′10.29″ N, 8°47′16.85″ W, 4 m a.s.l., growing in groups on *Q. robur* wood, 23 March 2021, *leg.* A. Mateos & S. De la Peña, AMI-SPL665. **Parque Natural Los Alcornocales.** Spain, Cádiz, Los Barrios, San Carlos del Tiradero, Parque Natural Los Alcornocales, 36°09′25.6″ N 5°34′56.3″ W, 210 m a.s.l., growing in groups on *Quercus canariensis* wood, 17 December 2022, *leg. A. Mateos*, *S. De la Peña & M. Plaza*, AMI-SPL1671.

***Xylaria xylarioides*** **(Speg.) Hladki & A.I. Romero, Fungal Diversity 42: 86 (2010)**

**Figure 27:**
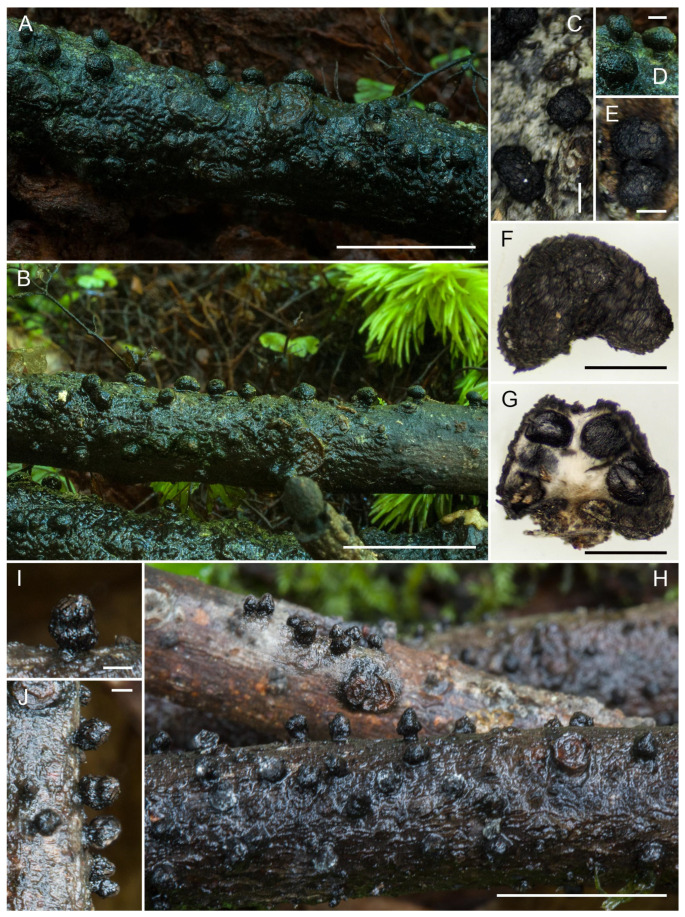
*Xylaria xylarioides*. Macromorphology. (**A**–**G**) AMI-SPL803. (**H**–**J**) AMI-SPL1971. (**A**,**B**) Stromata with morphological variability on the host natural substrate *Laurus novocanariensis* wood, in situ. (**C**–**E**,**I**,**J**) Subspherical or somewhat conical stroma with plaques or fissures and apical stripes. (**F**,**G**) Stroma exterior and interior in vertical section showing perithecia fully immersed in a white internal tissue. (**H**) Habit of mature and immature teleomorphic stromata on *Laurus nobilis* wood. Scale bars: (**A**,**B**,**H**) = 10 mm and (**C**–**G**,**I**,**J**) = 1 mm.

**Figure 28:**
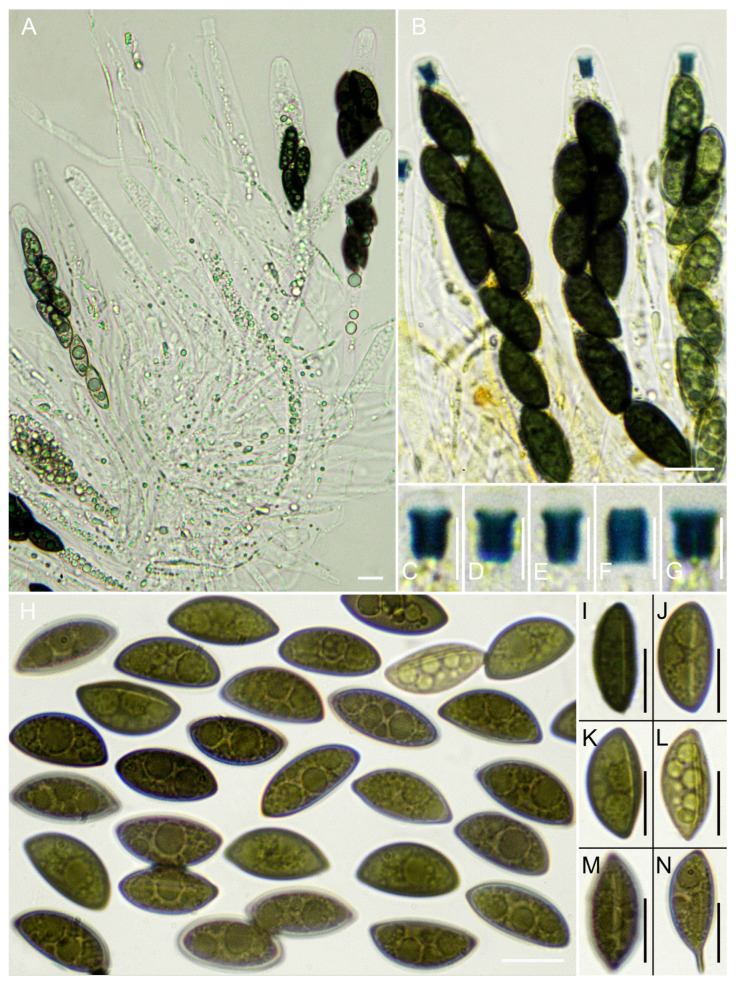
*Xylaria xylarioides*. Micromorphology. (**A**–**N**) AMI-SPL803. (**A**) Mature and immature asci with uniseriate to biseriate ascospores, in water. (**B**) Mature and immature asci, in IKI-2 reagent. (**C**–**G**) Ascal apical apparati in IKI-2 reagent. (**H**) Ascospores mature and immature, in water. (**I**–**N**) Ascospores in ventral view showing the germ slit, in water. Scale bars: (**A**,**B**,**H**–**L**) = 10 μm and (**C**–**G**) = 5 μm.

Figure 27 and Figure 28

**Teleomorph**. **Stromata** generally gregarious, sometimes solitary, simple, 1.6–2.5 mm total height, erect, sessile to shortly stipitate; fertile portion 1.3–1.7 mm high × 1.3–2.2 mm broad, subglobose or somewhat bilobulate, compressed, straight, with an obtuse or slightly conical apex, surface with somewhat circular, polygonal or irregular plaques or fissures, and some strips toward the apex, perithecial contours not exposed. Surface very rough, grayish brown to tan-brown, dark brown, becoming blackish; cortex leathery, up to 50(–70) μm thick, hard-textured, interior white, yellowish to grayish in the stipe region, homogeneous and solid. **Stipe** poorly defined, 0–0.6 mm high × 0–0.5 mm diam., terete, swollen at the base, surface black, leathery, with dense black stiff hairs. **Perithecia** immersed, spherical to subglobose, 550–650 μm high × 600–700 μm diam. **Ostioles** black, inconspicuous, with rounded papillae. **Asci** fusiform, (128.4–)135–178.4(–190) μm total length × 7.5–15(–18.2) μm wide, with a spore-bearing portion 90–110 μm long × 8–18.2 μm wide, containing eight ascospores arranged uniseriately or biseriately, apical apparatus urn-shaped to tubular, with a flat upper margin, (3.9–)4–4.4(–4.5) × (3–)3.1–3.6(–3.7) μm, euamyloid in Melzer’s reagent and Lugol’s solution. **Paraphyses** copious, filiform, thin-walled, equal to or longer than the asci, 2–3.5 μm wide at the base, tapering to 1.5–2.5 μm above the asci, somewhat septate, embedded in a gelatinous matrix, filled with oily guttules. **Ascospores** (15.5–)16.6–17.6–18.5(–20) × (7.1–)7.4–8.1–8.6(–9.5) μm, Q = (1.9–)2–2.2–2.4(–2.5), N = 35, Ve = 614 μm^3^, ellipsoid–inequilateral to navicular, with narrow subacute or slightly pinched ends, cellular appendage not observed, dark olivaceous to dark brown, smooth, guttulation variable (1–3 or more), germ slit straight or slightly curved, somewhat shorter than the spore length, located on the less convex side. Rarely, aberrant ascospores with one very sharply beaked end are observed.

**Notes**. *Xylaria xylarioides* produces small stromata with a very peculiar (penzigioid) morphology, although sometimes variable, which raises serious difficulties in distinguishing it from the species redescribed and recombined by Hladki and Romero [53], particularly because of stromatal shape, which may occasionally be conical and considerably larger, as well as when compared with similar species. In addition, the sequence identified as *X. apiculata* CBS 365.81 is recovered within the same lineage in our phylogenetic analyses, suggesting a very close relationship with *X. xylarioides*. However, a definitive assessment of its taxonomic status would require re-examination of the corresponding specimen and comparison with authenticated material.

*Xylaria globosa*, of comparable nodulose appearance due to deep wrinkles and furrows and likewise bearing sessile or sometimes stipitate stromata, is clearly distinct, especially in its ascospores, which are much larger in size and different in shape, and possess a slightly sigmoid germ slit [2,14,81].

It is also similar to *Xylaria papillata* and *Xylaria papillatoides*, from the Congo and Martinique, respectively; however, these taxa have stromata with a pointed central apex covered by a white outer layer, somewhat smaller ascospores with rounded ends, and uniseriate asci [2,10,19,20,82].

With respect to *X. arbuscula* var. *plenofissura*, the most discriminating character is ascospore shape, with narrower, slightly pinched ends in *X. xylarioides*, a distinction that agrees with Fournier et al. [10]. See also additional observations in the notes under *Xylaria acuminata* in this paper.

**Specimens examined. Cortegada Islands.** Spain, Galicia, Pontevedra, Parque Nacional de las Islas Atlánticas de Galicia, Cortegada Island, 42°36′56.1″ N 8°47′09.8″ W, 2 m a.s.l., growing in groups on *Laurus nobilis* wood, 30 November 2024, *leg.* A. Mateos & S. De la Peña, AMI-SPL1971; *ibid.*, AMI-SPL1973. **Madeira Islands.** Portugal, Madeira, São Vicente, Chão dos Louros, 32°45′37.1″ N, 17°00′58.5″ W, 820 m a.s.l., growing in groups on *Laurus novocanariensis* wood, 19 November 2021, *leg.* A. Mateos & S. De la Peña, AMI-SPL803; *ibid.*, AMI-SPL810.

## 4. Discussion

The present study substantially expands current knowledge of *Xylaria* diversity in Atlantic laurel forests by documenting at least 13 species from 83 collections across both insular and continental localities, namely, *X. acuminata*, *X. cinerea*, *X. conicoides*, *X. cylindracea*, *X. dactylata*, *X. hypoxylon*, *X. lauribaccicola*, *X. lauriphila*, *X. peritheciata*, *X. polyphaga*, *X. polymorpha*, *X. violaceorosea*, and *Xylaria xylarioides*. Several collections could not be successfully sequenced, and their taxonomic placement remains unresolved, suggesting that additional taxa may occur within the sampled material. Notably, eight species are newly described, highlighting the previously underestimated diversity of lignicolous fungi in these ecosystems. The high proportion of novel taxa detected within a relatively limited sampling effort indicates that Atlantic laurel forests harbor a rich but still poorly documented mycobiota, particularly within the Xylariaceae. Comparable patterns have been reported in other forest ecosystems, where targeted taxonomic surveys have revealed a high number of undescribed species within the genus [2,83].

Previous studies of *Xylaria* in Europe have largely focused on temperate forests or regional inventories (e.g., [6,7,9,10,11,65]), whereas Atlantic laurel forests have received relatively little attention despite being recognized as biodiversity hotspots [84,85]. Earlier records from Macaronesia and adjacent Atlantic regions included species such as *X. arbuscula*, *X. apiculata*, *X. polymorpha*, and *X. hypoxylon* [11,25,30], but the present results demonstrate that species richness in these habitats is substantially greater than previously documented.

The discovery of eight new species within a restricted geographic area is consistent with recent studies that have revealed extensive hidden diversity within the Xylariaceae. Integrative taxonomic approaches combining morphology with molecular phylogenetics have led to the description of numerous new taxa, particularly in tropical and subtropical forests [2,12,13,83]. These findings highlight the limitations of morphology-based species identification, as species diversity in *Xylaria* is often underestimated due to cryptic speciation and the relative morphological uniformity of stromata among closely related taxa.

A comparable trend has been observed in Atlantic laurel forests, where recent studies, including the present study, have documented a considerable number of previously undescribed fungal taxa across multiple lineages, including the establishment of new genera and more than 20 new species, many associated with lignicolous or foliar substrates [86,87,88,89,90]. Together, these results reinforce the view that fungal diversity in these ecosystems remains significantly underestimated.

In this study, multilocus phylogenetic analyses proved essential for species delimitation. Early molecular studies of *Xylaria* relied primarily on the internal transcribed spacer (ITS) region [31,32,34], which often lacks sufficient resolution to distinguish closely related taxa. The inclusion of additional loci, such as LSU, *RPB2*, and *TUB2*, has been shown to improve phylogenetic resolution within the Xylariaceae [35,38]. Despite incomplete sequencing for some specimens, the multilocus approach employed here allowed the recognition of well-supported clades corresponding to the newly described species, providing robust evidence for their taxonomic independence.

From an ecological perspective, species of *Xylaria* are important components of forest ecosystems, contributing to the decomposition of lignocellulosic substrates and nutrient cycling [5,83]. The environmental conditions characteristic of Atlantic laurel forests—high humidity, relatively stable temperatures, and abundant woody substrates—likely favor the development of lignicolous fungi. Moreover, several *Xylaria* species are known to occur as endophytes in living plant tissues, producing fruiting bodies during the saprobic phase following host senescence [5,34]. Such life-history strategies may facilitate persistence and diversification in long-term stable habitats such as Macaronesian laurel forests.

Biogeographically, Atlantic laurel forests are considered remnants of the subtropical forests that covered large areas of southern Europe during the Tertiary [84]. These ecosystems have acted as refugia for numerous plant and animal lineages and likely play a similar role for fungi. The discovery of several previously unknown *Xylaria* species supports the hypothesis that laurisilva ecosystems represent important centers of fungal diversity and potentially endemism. However, broader sampling across Macaronesia and continental refugial areas will be necessary to clarify distribution patterns and evolutionary relationships.

Overall, this study highlights the importance of integrative taxonomy in uncovering fungal diversity in understudied ecosystems. Further research combining extensive field sampling, detailed morphological analyses, and multilocus phylogenetics will be essential to improve our understanding of the diversity, ecology, and biogeography of *Xylaria* and other lignicolous fungi. The high number of undescribed species detected from a relatively limited sampling effort suggests that current estimates of fungal diversity in Atlantic laurel forests, and likely in other structurally complex, humid ecosystems, remain far from complete. This underestimation is further exacerbated by sampling biases and the prevalence of cryptic taxa that are difficult to detect using traditional approaches [14,83].

These findings emphasize that Atlantic laurel forests should be regarded not only as refugia of relict plant diversity, but also as reservoirs of previously unrecognized fungal lineages, whose study is crucial for a comprehensive understanding of biodiversity patterns in European forest ecosystems.

## Figures and Tables

**Figure 11:**
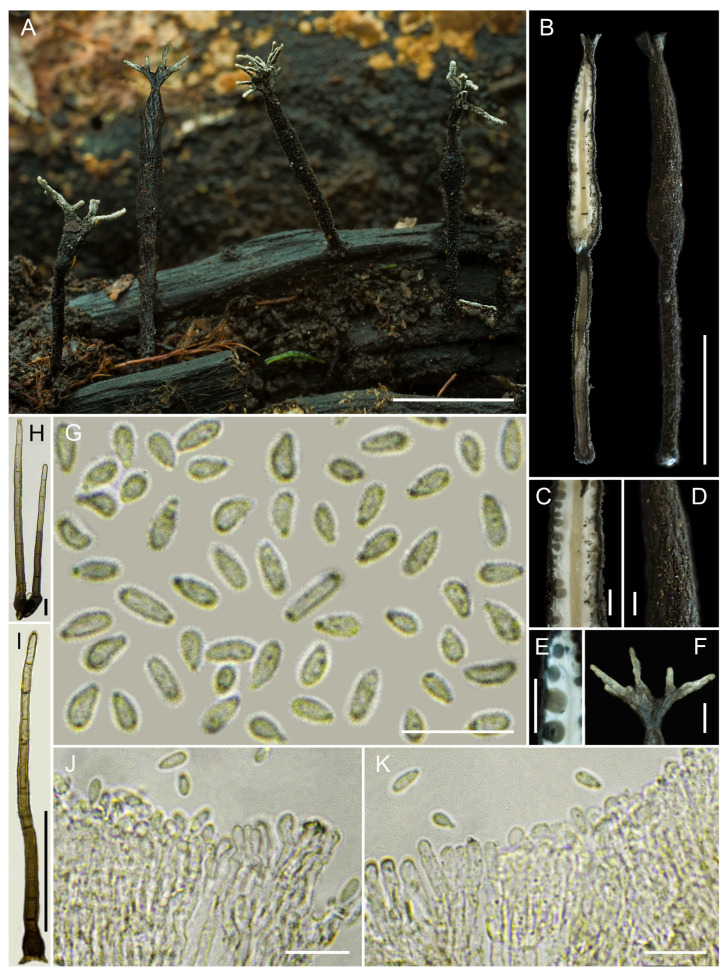
*Xylaria dactylata* macro- and micro-morphology. (**A**–**K**) AMI-SPL761. (**A**) Immature stromata, on host surface of *Laurus novocanariensis*, in situ. (**B**) Stroma exterior and interior in vertical section. (**C**,**E**) Stroma in vertical section showing perithecia fully immersed in a white fibrous internal tissue. (**D**) Stroma showing gray superficial stripes. (**F**) Anamorphic stroma. (**G**) Conidia, in water. (**H**,**I**) Hairy surface, hairy tomentose. (**J**,**K**) Palisade-like conidiophores with a conidiogenous cell. Scale bars: (**A**,**B**) = 10 mm, (**C**–**F**) = 1 mm, (**G**–**K**) = 10 μm, and (**I**) = 50 μm.

**Table 1:** Sampling site (SS) details, including geographical information, protection status, and nature 2000 classification. Localities (P: Portugal; S: Spain). Coordinates (WGS84; Lat.: latitude; Lon.: longitude). Altitude (m a.s.l.: meters above sea level). SD: sampling date.

SS	Localities	Lat.	Lon.	Alt.	SD
SS01	Azores (P). Terceira. Angra do Heroísmo, Terra-Chã, Matela de Baixo	38.700	−27.259	470	12 January 2022
SS02	Azores (P). Terceira. Angra do Heroísmo, Serra de Sta. Bárbara	38.727	−27.328	866	13 January 2022
SS03	Azores (P). Terceira. Angra do Heroísmo, Algar do Carvão, Terra Brava	38.736	−27.201	670	14 January 2022
SS04	Madeira (P). Madeira. Ribeiro Frio	32.737	−16.886	940	18 November 2021
SS05	Madeira (P). Madeira. Ribeira da Janela	32.816	−17.148	1100	19 November 2021
SS06	Madeira (P). Madeira. São Vicente, Chão dos Louros	32.760	−17.016	820	20 November 2021
SS07	Canary Islands (S). Tenerife. Mirador del Escobón, Anaga	28.538	−16.300	660	3 December 2021
SS08	Canary Islands (S). Tenerife. Monte del Agua, Teno	28.329	−16.810	990	4 December 2021
SS09	Canary Islands (S). La Gomera. Garajonay	28.110	−17.265	1470	5 December 2021
SS10	Sierra de las Villuercas (S). Cáceres. Alía, Lorera de la Trucha, Garganta de la Trucha	39.547	−5.249	640	16 January 2023
SS11	Sierra de las Villuercas (S). Cáceres. Alía, Lorera de la Trucha, Garganta de la Trucha	39.547	−5.249	640	16 January 2023
SS12	Sierra de las Villuercas (S). Cáceres. Villar del Pedroso, Lorera del Mesto, Garganta del Mesto, Valle del Hospital del Obispo	39.590	−5.326	770	17 January 2023
SS13	Peneda-Gerês (P). Viana do Castelo. Arcos de Valdevez, Sistelo, Peneda-Gerês	41.954	−8.408	260	9 November 2022
SS14	Peneda-Gerês (P). Braga. Terras de Bouro, Freitas, Peneda-Gerês	41.704	−8.217	400	9 November 2022
SS15	Peneda-Gerês (P). Viana do Castelo. Arcos de Valdevez, Sistelo, Ponte de Cabreiro, Peneda-Gerês National Park	41.738	−8.501	180	10 November 2022
SS16	Atlantic Islands of Galicia (S). Cortegada. Pontevedra, Atlantic Islands of Galicia	42.620	−8.788	4	9 November 2022
SS17	Atlantic Islands of Galicia (S). Cortegada. Pontevedra, Atlantic Islands of Galicia	42.616	−8.785	2	9 November 2022
SS18	Atlantic Islands of Galicia (S). Cortegada. Pontevedra, Atlantic Islands of Galicia	42.616	−8.787	4	10 November 2022

**Table 2:** List of species, specimens, and gene sequence accession numbers used in this study. New sequences provided in this study are in bold. “NA”: not available.

Taxon	Substrate/Country	Voucher/Isolate	GenBank Accession Number			
			ITS	LSU	RPB2	TUB
***Xylaria acuminata*** **A. Mateos & De la Peña-Lastra, sp. nov.**	Wood/Portugal: Azores	AMI-SPL1339	PZ348472	NA	NA	PZ334375
***Xylaria acuminata*** **A. Mateos & De la Peña-Lastra, sp. nov.**	Wood/Portugal: Azores	AMI-SPL1344	PZ348473	NA	NA	NA
*Xylaria apiculata* Cooke	Broadleaved bark/Spain	ERD-7166	MN644466	MN644468	NA	NA
*Xylaria apiculata* Cooke	Colombia	CBS 365.81	AF163027	NA	NA	NA
*Xylaria arbuscula* Sacc.	Germany	CBS:126415	KY610394	MH875560	KY624287	KX271257
*Xylaria arbuscula* Sacc.	Bark/Taiwan	89041211	GU300090	NA	NA	NA
*Xylaria arbuscula* Sacc.	NA	FL1030	OQ831957	NA	KU684247	KU684165
*Xylaria arbuscula* var. *plenofissura* Y.M. Ju & Tzean	Taiwan	HAST 93082814	GU339495	NA	GQ844804	GQ478225
*Xylaria bambusicola* Y.M. Ju & J.D. Rogers	Taiwan	WSP205	NR_153200	NA	NA	NA
*Xylaria bambusicola* Y.M. Ju & J.D. Rogers	Bamboo culm/Thailand	JDR162	GU300088	NA	GQ844801	GQ478223
*Xylaria carpophila* (Pers.) Fr.	Germany	M:M-0125884	AM993124	NA	NA	NA
*Xylaria cinerea* J. Fourn. & M. Stadler	Spain	MUCL:51825/agrMS224	FN689805	NA	NA	NA
*Xylaria cinerea* J. Fourn. & M. Stadler	Corticated branch/France	MUCL:51696/agrMS206	FN689799	NA	NA	NA
***Xylaria cinerea*** **J. Fourn. & M. Stadler**	**Wood/Spain: Cortegada**	**AMI-SPL673**	PZ348458	NA	PZ369694	PZ334368
***Xylaria cinerea*** **J. Fourn. & M. Stadler**	**Wood/Portugal: Azores**	**AMI-SPL1341**	PZ348455	NA	NA	NA
***Xylaria cinerea*** **J. Fourn. & M. Stadler**	**Wood/Spain: Cortegada**	**AMI-SPL2168**	PZ348456	NA	NA	NA
***Xylaria cinerea*** **J. Fourn. & M. Stadler**	**Wood/Spain: Canarias**	**AMI-SPL957**	PZ348457	NA	PZ369693	PZ334367
*Xylaria coccophora* Mont.	Wood/French Guiana	786	GU300093	NA	NA	NA
*Xylaria comosa* (Mont.) Dennis	French Guiana	F-GUY-12-094	MF038919	NA	NA	NA
*Xylaria comosa* (Mont.) Dennis	French Guiana	F-GUY-12-050	MK546718	NA	NA	NA
***Xylaria conicoides*** **A. Mateos & De la Peña-Lastra, sp. nov.**	Wood/Portugal: Madeira	AMI-SPL769	PZ348474	NA	PZ369701	PZ334379
*Xylaria corniformis* (Fr.) Fr.	*Fagus sylvatica*/Germany	NW-FVA5285	MT561412	NA	NA	NA
*Xylaria crozonensis* (Pers.) Grev.	Bark/France	398	GU324748	NA	GQ848361	GQ502697
*Xylaria cubensis* (Mont.) Fr.	?	4355-2	PQ632313	NA	NA	NA
***Xylaria cylindracea* A. Mateos & De la Peña-Lastra, sp. nov.**	Wood/Portugal: Azores	**AMI-SPL1342**	PZ348478	NA	NA	PZ334378
***Xylaria dactylata*** **De la Peña-Lastra & A. Mateos, sp. nov.**	Wood/Portugal: Madeira	AMI-SPL761	PZ348479	PZ348487	NA	PZ334380
***Xylaria dactylata*** **De la Peña-Lastra & A. Mateos, sp. nov.**	Wood/Portugal: Madeira	AMI-SPL761-B	PZ348480	NA	NA	NA
*Xylaria delitschii* Auersw.	Subterranean fruit/Germany	cult_07_X2	HQ414586	NA	NA	NA
*Xylaria filiformis* (Alb. & Schwein.) Fr.	Leaves or petioles/China	186	MF774332	NA	NA	NA
*Xylaria filiformis* (Alb. & Schwein.) Fr.	Herbaceous stem/Iran	?	KP218907	NA	NA	NA
*Xylaria hypoxylon* (L.) Grev.	Sweden	CBS:122620	KY610407	KY610495	KY624231	KX271279
*Xylaria hypoxylon* (L.) Grev.	Wood/Belgium	CBS:152	GU300096	NA	NA	NA
***Xylaria hypoxylon*** **(L.) Grev.**	**Wood/Spain: Canarias**	**AMI-SPL1139**	PZ348459	NA	NA	NA
*Xylaria hypoxylon* (L.) Grev.	Wood/Taiwan	HAST95082001	GU300095	NA	GQ844811	GQ487703
*Xylaria karsticola* J. Fournier & M. Stadler	Buried stump/France	MUCL:47969/agrMS212	FN689803	NA	NA	NA
*Xylaria karsticola* J. Fournier & M. Stadler	Buried stump/France	MUCL:51605/agrMS210	FN689802	NA	NA	NA
***Xylaria lauribaccicola*** **De la Peña-Lastra & A. Mateos, sp. nov.**	Fruit/Spain: Canary Islands	AMI-SPL1098	PZ348471	NA	PZ369700	PZ334374
***Xylaria lauriphila* De la Peña-Lastra & A. Mateos, sp. nov.**	Wood/Spain: Canary Islands	AMI-SPL1119	PZ348475	NA	PZ369702	PZ334376
***Xylaria lauriphila* De la Peña-Lastra & A. Mateos, sp. nov.**	Wood/Spain: Canary Islands	AMI-SPL1121	PZ348476	NA	PZ369703	PZ334377
***Xylaria lauriphila* De la Peña-Lastra & A. Mateos, sp. nov.**	Wood/Spain: Canary Islands	AMI-SPL1833	PZ348477	NA	NA	NA
*Xylaria longipes* Nitschke.	Leaf/China	S2L26	PQ584562	NA	NA	NA
*Xylaria longipes* Nitschke.	Ukraine	IBK 2726	PP830543	NA	NA	NA
*Xylaria longipes* Nitschke.	Angiosperm wood/Germany	CBS 147.73	AY909017	NA	NA	NA
*Xylaria longipes* Nitschke.	Germany	CBS 580.88	AY909015	NA	NA	NA
*Xylaria oligotoma* Sacc. & Paol.	Dead wood/French Guiana	784	GU300092	NA	NA	NA
*Xylaria oxyacanthae* Tul. & C. Tul.	On seeds/China	FCATAS906/132	MZ620655	MZ703200	MZ678636	MZ695790
*Xylaria oxyacanthae* Tul. & C. Tul.	Subterranean fruit/Germany	cult_07_X3	HQ414587	NA	NA	NA
***Xylaria peritheciata*** **A. Mateos & De la Peña-Lastra, sp. nov.**	**Wood/Portugal: Azores**	**AMI-SPL1256**	PZ348468	NA	NA	PZ334373
***Xylaria peritheciata*** **A. Mateos & De la Peña-Lastra, sp. nov.**	**Wood/Portugal: Azores**	**AMI-SPL1258**	PZ348469	PZ348486	NA	NA
***Xylaria peritheciata*** **A. Mateos & De la Peña-Lastra, sp. nov.**	**Wood/Portugal: Azores**	**AMI-SPL1280**	PZ348470	NA	PZ369699	NA
*Xylaria polymorpha* (Pers.) Grev.	France	MUCL:49884	KY610408	KY610464	KY624288	KX271280
*Xylaria polymorpha* (Pers.) Grev.	France	MUCL:49904/agrMS243	FN689809	NA	NA	NA
***Xylaria polymorpha*** **(Pers.) Grev.**	**Wood/Spain: Cortegada**	**AMI-SPL1586**	PZ359624	NA	NA	NA
***Xylaria polyphaga*** **De la Peña-Lastra & A. Mateos, sp. nov.**	**Seeds/Spain: Cortegada**	**AMI-SPL1167**	PZ348462	PZ348482	NA	NA
***Xylaria polyphaga*** **De la Peña-Lastra & A. Mateos, sp. nov.**	**Wood/Spain: Cortegada**	**AMI-SPL2003**	PZ348463	NA	NA	NA
***Xylaria polyphaga*** **De la Peña-Lastra & A. Mateos, sp. nov.**	**Seeds/Portugal: Azores**	**AMI-SPL1254**	PZ348464	PZ348483	PZ369697	PZ334369
***Xylaria polyphaga*** **De la Peña-Lastra & A. Mateos, sp. nov.**	**Wood/Portugal: Azores**	**AMI-SPL1257**	PZ348465	PZ348484	NA	NA
***Xylaria polyphaga*** **De la Peña-Lastra & A. Mateos, sp. nov.**	**Wood/Spain: Cádiz**	**AMI-SPL1657**	PZ348466	NA	NA	NA
***Xylaria polyphaga*** **De la Peña-Lastra & A. Mateos, sp. nov.**	**Wood/Spain: Canary Islands**	**AMI-SPL925**	PZ348467	PZ348485	PZ369698	PZ334372
*Xylaria primorskensis* Y.M. Ju, H.M. Hsieh, Lar.N. Vassiljeva & Akulov	USA	F-779951 (UPS)	MH782087	NA	NA	NA
*Xylaria primorskensis* Y.M. Ju, H.M. Hsieh, Lar.N. Vassiljeva & Akulov	Russia	478	FJ707473	NA	NA	NA
*Xylaria sicula* Passerini & Beltrani	Fallen leaves/Croatia	CNF 2/11087	OQ865227	OQ865230	OQ877112	OQ877123
*Xylaria sicula* Passerini & Beltrani	Fallen leaves/Taiwan	90071613	GU300081	NA	NA	NA
*Xylaria vasconica*	France	MUCL:51705/agrMS222	FN689804	NA	NA	NA
*Xylaria vasconica*	USA	MUCL:51697/agrMS207	FN689800	NA	NA	NA
*Xylaria venosula* Speg.	USA: Hawaiian Islands	YMJ94080508	EF026149	NA	NA	EF025617
*Xylaria venosula* Speg.	Root/China	MFLUCC21-0015	MZ463136	MZ463179	MZ970704	MZ998955
*Xylaria venosula* Speg.	Decaying branches/China	CC.BYG51.1	PP407909	PP407722	NA	NA
*Xylaria venosula* Speg.	Decaying twig/India	PUFNI1763	MZ292933	NA	NA	NA
*Xylaria venustula* Sacc.	Bark/Taiwan	88113002	GU300091	NA	NA	NA
***Xylaria violaceorosea***	**Wood/Spain: Cortegada**	**AMI-SPL1479**	PZ348461	NA	NA	NA
*Xylaria xylarioides* Speg.	Acer pseudoplatanus/Spain	ERD-9783	OR536215	NA	NA	NA
*Xylaria xylarioides* Speg.	Wood/Iran	GUM<IRN>:1051	KP218909	NA	NA	NA
***Xylaria xylarioides*** **Speg.**	**Wood/Portugal: Madeira**	**AMI-SPL803**	PZ348460	NA	PZ369695	PZ334370
***Xylaria xylarioides*** **Speg.**	**Wood/Spain: Cortegada**	**AMI-SPL1971**	PZ348461	NA	PZ369696	PZ334371
*Biscougnauxia arima* F.San Martín Y.M. Ju & J.D. Rogers	Wood/Mexico	BCRC34030	EF026150	PQ215672	GQ304736	AY951672
*Biscougnauxia*_mediterranea (De Not.) Kuntze	Corticated wood/France	BCRC34037	EF026134	NA	GQ844765	AY951684

## Data Availability

The data presented in this study are openly available in [ZENODO] at [https://doi.org/10.5281/zenodo.20001511].

## Supplementary Materials

The following supporting information can be downloaded at: https://www.mdpi.com/article/10.3390/life16060993/s1, Table S1. Comparative morphological and ecological characters of the newly described species recovered within the *Xylaria polyphaga*–*X. lauriphila* lineage.
