# Supplementary material for: Diversity of the Genus Xylaria in European Atlantic Lauroid Forest: New Records and Description of Eight New Species
Source: Life (Basel). 2026 Jun 12;16(6):993. doi: 10.3390/life16060993 (PMC13302140; doi:10.3390/life16060993)
Supplement: Supplementary file 1 [file life-16-00993-s001.zip › life-4347886-supplementary.pdf]

Table S1. Comparative morphological and ecological characters of the newly described species recovered within the *Xylaria polyphaga*–*X. lauriphila* lineage.

**Character**	***X. polyphaga***	***X. conicoides***	***X. lauribaccicola***	***X. lauriphila***
Substrate	Wood and fruits of several hosts (*Laurus*, *Eucalyptus*)	Wood of *Ocotea foetens*	Fruits of *Laurus novocanariensis*	Wood of *Laurus* spp.
Total stromata [illegible]	(8–)15–20(–27) mm	6–10(–11) mm	30–46 mm	10.5–14(–15) mm
Stipe length	4–11 mm	2–4 mm	(8–)20–29(–32) mm	3–5 mm
Stromatal habit	Subcylindrical to narrowly fusiform	Conical to fusiform	Variable, twisted, sometimes furcate	Subcylindrical to fusiform
Sterile apex	1–2.5 mm, rounded or mucronate	0.5–1.2 mm, distinctly conical	1–2 mm, mucronate-apiculate	1.2–1.8 mm, mucronate-apiculate
Perithecial contours	Not to faintly exposed	Not exposed	Strongly exposed at maturity	Slightly to distinctly exposed
Stromatal surface	Longitudinal scales, strips and plates	Broad carbonaceous strips, grey-silvery bands	Grey striate strips delimiting pale fissures	Brownish or pinkish strips/plaques
Internal tissue	White with ochraceous inner core	White, becoming pinkish-grey	White to pinkish cream	White to cream, yellowish [illegible]
Apical apparatus	2.2–3.5 × 3.5–4 µm	3.4–4.3 × 2–2.7 µm	4.9–6.3 × 2.5–3.3 µm	4.6–5.4 × 3–3.3 µm
Ascospore size	13.8 × 5.8 µm	15.5 × 5.8 µm	18.1 × 7.7 µm	17.6 × 6.2 µm
Ascospore volume	255 μm^3	271 μm^3	560 μm^3	358 μm^3
Ascospore ends	Broadly rounded	Rounded or one acute	Narrowly rounded to acute	Rounded to slightly pinched
Germ slit	¾ spore length	4/5 to almost entire length	About 4/5 length	Slightly shorter than spore length
Geographic distribution	Azores, Canary Islands, Cortegada, Cádiz	Madeira	La Gomera	La Gomera, Peneda-Gerês
